# A core outcome set for research on masculinizing genital gender-affirming surgery: international consensus results from the GenderCOS project

**DOI:** 10.1016/j.eclinm.2025.103325

**Published:** 2025-07-01

**Authors:** Philippine J. Roijer, Marleen S. Vallinga, Thomas E. Pidgeon, Matteo Angelini, Aline Ceulemans, Alex Bakker, Brenda Carrière, Tina Rashid, James Bellringer, Javier Belinky, Marlon Buncamper, Shane D. Morrison, Walter P. Bouman, Tim C. van de Grift, Mark-Bram Bouman, Margriet G. Mullender

**Affiliations:** a Amsterdam UMC (Location VUmc) - Amsterdam University Medical Centre, Department of Plastic, Reconstructive and Hand Surgery, Amsterdam, the Netherlands; b Amsterdam Public Health Research Institute, Amsterdam, the Netherlands; c Russells Hall Hospital, Dudley Group Foundation Trust, Department of Plastic Surgery, Dudley, United Kingdom; d Vita-Salute San Raffaele University, IRCCS San Raffaele Hospital, Milan, Italy; e University Hospital Ghent, Department of Plastic Surgery, Ghent, Belgium; f Independent Scholar, Utrecht/‘s Hertogenbosch, the Netherlands; g Chelsea Centre for Gender Surgery (CCGS), Chelsea and Westminster Hospital NHS Trust, London, United Kingdom; h Nuffield Health Parkside Hospital, London, United Kingdom; i Guemes Hospital and Urological Center CDU, Buenos Aires, Argentina; j Division of Plastic and Reconstructive Surgery, Department of Surgery, University of Washington, Seattle, WA, USA; k Department of Urology, University of Washington, Seattle, WA, USA; l Nottingham Centre for Transgender Health, Nottingham, United Kingdom; m Department of Psychiatry and Medical Psychology, Zaans Medical Center, Zaandam, the Netherlands

**Keywords:** Core outcome set, Delphi, Consensus, Transgender, Gender diverse, Masculinizing genital gender-affirming surgery

## Abstract

**Background:**

Masculinizing genital gender-affirming surgery (gGAS) for transgender and gender diverse people encompasses a complex, heterogenous, and modular set of procedures. To enable evidence-based decision-making or meta-analysis, a uniformity of outcome research is needed. Currently, this is hindered by non-standardized outcome reporting. This study aims to develop a core outcome set (COS) for research on masculinizing gGAS to address this issue.

**Methods:**

An international, multidisciplinary study steering group was established, consisting of 16 professional experts (PE) and lived experience experts (LEE), to advise on and support the development of this COS. The steering group convened online on 16 June 2022, 28 September 2023, 2 May 2024, and 29 August 2024. Potential outcomes were identified through interviews and focus groups with LEE and a systematic literature review convened up to September 2023. LEE and PE were recruited globally via websites, social platforms, scientific meetings, patient organizations, and posters and invited to participate in three e-Delphi survey rounds. In the first round, participants rated each outcome's importance on a 5-point Likert scale and provided reasoning for their rating. In subsequent rounds, participants re-rated the outcomes using illustrative quotes and rankings based on prior ratings to refine consensus. Pre-defined criteria guided outcome inclusion and exclusion. A final online consensus meeting with LEE and PE finalized the COS.

**Findings:**

Initial outcome gathering identified 384 unique outcomes, of which 38 outcomes were selected for the e-Delphi survey rounds. Thirty-two LEE and 52 PE participants, most from Europe, the UK, and the USA, completed all three survey rounds (February, May, and July 2024). Final consensus was reached in September 2024 on including one clinical outcome, three adverse events, and six patient-reported outcomes in the COS for masculinizing gGAS. The core outcomes are sensibility in the neo-phallus, additional surgery, ability to achieve orgasm, sexual well-being, and satisfaction with neo-genital aesthetics. For urethral lengthening, additional core outcomes include neo-urethral fistula, stricture, and the ability to void while standing. For phalloplasty, additional outcomes are flap necrosis and donor site morbidity.

**Interpretation:**

Developing a COS for masculinizing gGAS is a crucial step towards standardized outcome measurement and reporting in clinical research. Implementation could facilitate data comparison, meta-analysis and evidence-based guideline development, ultimately improving personalized surgical care.

**Funding:**

None received.

**Research in context**

**Evidence before this study**

Before the start of the development of a Core Outcome Set (COS) for masculinizing genital gender-affirming surgery (gGAS), PubMed and COMET databases were searched using terms (and equivalents) “Core Outcome Set” AND “gender-affirming surgery” OR “transgender” OR “gender diverse” (with no time limits, in July 2021), and to our knowledge, no study reported on the development of a COS in masculinizing gGAS. To identify all outcomes reported after masculinizing gGAS, a comprehensive systematic review was performed as a COS development step, demonstrating heterogeneous outcome reporting and confirmed the need for standardization within the field. This study aimed to develop a COS to achieve consistency across masculinizing gGAS research.

**Added value of this study**

This study describes key stakeholder (i.e., international Lived Experience Experts (LEE) and multidisciplinary Professional Experts (PE)) consensus on ten outcomes to consistently measure and report in masculinizing gGAS research. Deliberative consensus methods were employed, including three rounds of e-Delphi surveys and a consensus meeting. This study explored which outcomes were deemed most important and why, and PE agreement was reached on the definitions of the outcomes included in the core set.

**Implications of all the available evidence**

This COS can improve data consistency in masculinizing gGAS research, enabling proper data comparison and meta-analysis. This standardization can contribute to a robust evidence-base, supporting surgical decision-making and guideline development. While the current study reached international key stakeholder consensus on what to measure, future steps will involve agreeing on how and when the core outcomes should be measured.

## Introduction

In recent years, there has been a steady increase in the number of transgender and gender diverse individuals seeking masculinizing genital gender-affirming surgery (gGAS).^1^ This increase coincides with advancements in surgical techniques and the growing expertise of surgeons able to perform complex genital reconstruction.^2^ Moreover, there is an expanding body of literature on masculinizing gGAS.^3^^,^^4^

Current masculinizing gGAS procedures include the creation of a phallus, with or without urethral lengthening (i.e., phalloplasty or metoidioplasty), a scrotum (i.e., scrotoplasty), and a corona (i.e., coronaplasty). These procedures are performed using a variety of techniques, including different skin flaps from a range of donor sites. Similarly, distinct surgical methods are employed, such as staged procedures, skin flap pre-expansion, and varying numbers of vascular anastomoses and/or nerve coaptations.^3^ Deciding on the most appropriate procedure involves the patient, a mental health professional, and the surgeon, preferably through shared decision-making. The surgeon's options are typically based on their training, experience, personal preferences, and the patient's specific physical characteristics. The diversity of surgical procedures and techniques, along with the absence of internationally recognized standards for surgical training and certification, gives rise to considerable practice variation in the field of masculinizing gGAS.

While there is a growing body of literature on masculinizing gGAS, the evidence is generally of low quality. This is due to lack of generalizability, predominantly retrospective study designs, short follow-up periods, and significant variability in how outcomes are measured and reported.^3^^,^^4^ Additionally, patient-reported outcomes are often underreported, and frequently rely on non-validated and non-population specific measurement tools. The inconsistency in reporting across studies limits meta-analyses or comparative effectiveness studies, which routinely offer avenues for more robust evidence-base.

Guideline and policy development, as well as informed (shared) decision-making by transgender and gender diverse individuals and their healthcare providers, can be limited when the evidence-base is insufficient to adequately inform decision making.^5^ To advance the field of masculinizing gGAS, comparative and standardized research is needed. A Core Outcome Set (COS) facilitates this by establishing an international, multi-stakeholder consensus on a minimum set of outcomes that should be measured uniformly and reported consistently in all studies related to a specific health condition, area, or intervention.^6^ The GenderCOS project was designed to develop a COS for both feminizing and masculinizing gGAS research.^7^ The development of a COS for masculinizing gGAS is described in this paper.

## Methods

The GenderCOS project started in September 2021. An international study steering group was formed with multidisciplinary professional experts in the field of gender-affirming genital surgery and lived experience experts (i.e., transgender and gender diverse individuals who have had gGAS). Steering group members were based in Europe, the United Kingdom, North America, and South America. Together with the study management group, responsible for daily management, they collaboratively designed and executed the project. All members of the study steering group are listed as authors on this manuscript. The steering group convened in four online meetings, held on 16 June 2022, 28 September 2023, 2 May 2024, and 29 August 2024. Between these meetings, the study management group provided regular updates and maintained communication via email. The COS was established in September 2024.

### Protocol/Registry entry

This study was prospectively registered in the Core Outcome Measures in Effectiveness Trials (COMET) database under study number 2067.^8^ A study protocol describing the GenderCOS project was published prior to the start of the consensus process.^7^ The study methodology was designed based on the COMET Handbook, using the Core Outcome Set-STAndards for Development (COS-STAD) recommendations.^6^^,^^9^ A systematic review was conducted as part of the project and registered in the International Prospective Register of Systematic Reviews (PROSPERO) under number CRD42022347400.^10^

### Participants

The study steering group identified two relevant stakeholder groups that should be included in the study: Lived Experience Experts (LEE) and Professional Experts (PE). Eligible LEE participants included transgender and gender diverse individuals who had undergone masculinizing gGAS (i.e., phalloplasty or metoidioplasty with or without urethral lengthening, scrotoplasty, and coronaplasty) at least three months prior to participation. Professional Experts (PE) comprised various professionals involved in masculinizing gGAS. This group included surgeons (e.g., plastic surgeons, urologists and gynecologists), other healthcare providers involved in masculinizing gGAS-counseling or care (such as psychologists, physiotherapists, sexologists, physician assistants, psychiatrists, endocrinologists, therapists, and social workers), as well as researchers in the field. All participants needed to be proficient in English, Spanish, or Dutch and be able to provide an informed e-consent.

Participants were recruited through various channels, including websites; online social platforms (Instagram and LinkedIn); scientific meetings (World Professional Association of Transgender Health (WPATH), European Professional Association of Transgender Health (EPATH), European Society for Sexual Medicine (ESSM), and Gender Rounds); patient organizations around the world; the study steering group's international network; posters displayed in healthcare facility waiting areas; and WPATH e-blasts. Participants registered for participation on the GenderCOS website using an email address, and they received all further communication via email. Prior to participation, participants could review an information letter and were required to give informed e-consent through the survey software.

### Procedures

**Fig. 1:**
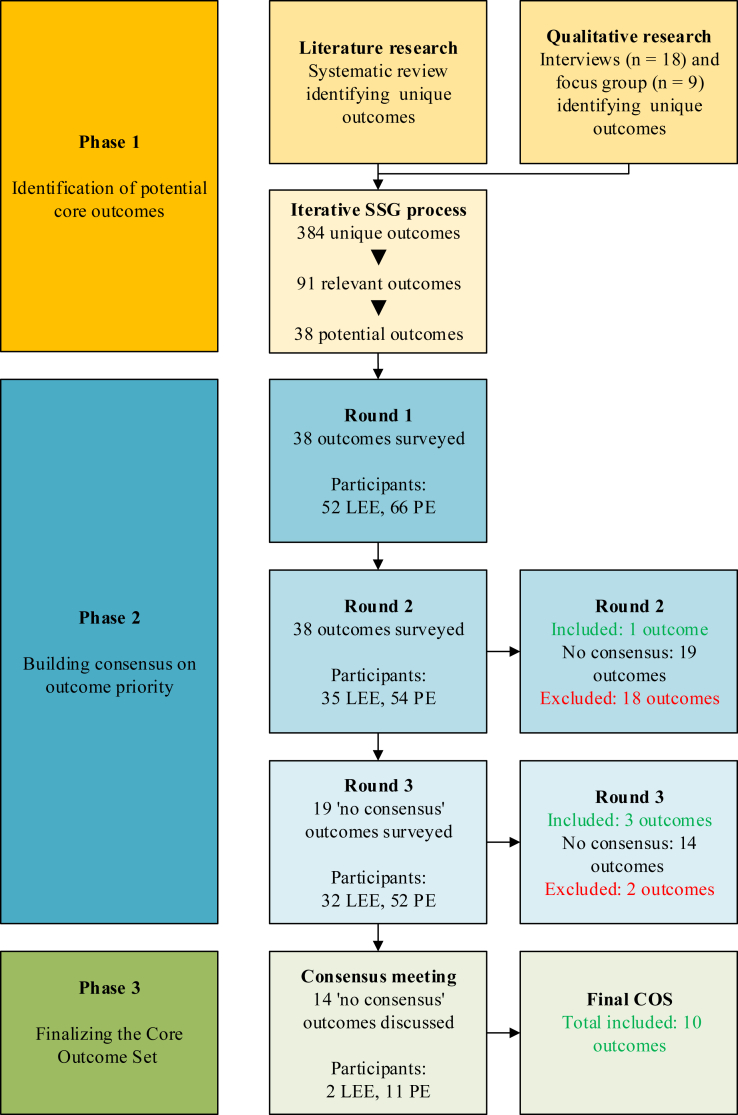
Overview of study phases and results. SSG, Study Steering Group; LEE, Lived Experience Expert; PE, Professional Expert; COS, Core Outcome Set.

The development of the COS followed three phases: 1) the identification of all relevant outcomes and the establishment of a list of potential core outcomes, 2) a 3 round Delphi process to build consensus among stakeholders on what they considered to be critically important outcomes, and 3) a consensus meeting to reach a final consensus on those outcomes that had not yet been decided. An overview of the development phases can be found in Fig. 1.

### Phase 1: identification of potential core outcomes

A systematic review of outcomes reported following masculinizing gGAS was conducted in September 2023 and published in November 2024 as a part of this project.^3^ The review identified individual outcomes, which were then condensed based on similar spelling and meaning by the study management group, resulting in a list of unique outcomes.

Prior to this project, interviews and focus groups were conducted with transgender and gender diverse individuals to gain insight into why they chose specific surgical options and what outcomes they considered important. This qualitative data was collected to help develop a decision aid and was reused in the GenderCOS project, as it addressed a similar research question.^11^ The study management group extracted all unique outcomes mentioned in the interview and focus group transcripts.

A structured process was used to select potential core outcomes from the extensive list of outcomes to be included in the Delphi process.^12^ Initially, the study management group categorized outcomes into three groups: red (low relevance, redundant, or obsolete), orange (moderate relevance), and green (high relevance, frequently reported, and mentioned in qualitative data). The study steering group then prioritized the green and orange outcomes using Google Forms, and checked if they agreed with exclusion of the red outcomes. Finally, the results were discussed in an online study steering group meeting where a nominal group technique was used to determine the final list of outcomes to be included in the Delphi process.^12^

The study management group used the data from the systematic review to formulate definitions for each of these outcomes. After review by the study steering group, the definitions were finalized for further consensus during Phase 2.

### Phase 2: building consensus on outcome priority

An e-Delphi procedure was employed to build consensus on the priority of outcomes. Surveys were administered online using LimeSurvey software (version 2.6.7 Build Sondages Pro 1.7.3.). The surveys were available in English, Spanish, and Dutch. In each round, participants had 6 weeks to complete the survey. Up to 6 reminders were sent to (registered) participants during this time. The analysis and the subsequent survey preparation were conducted between survey rounds and required a maximum of 6 weeks.

### Statistics

The survey responses of each round were descriptively analyzed using SPSS (version 28).

### Round 1

In the first survey round, LEE and PE were asked to rate the importance of each outcome on a 5-point Likert scale (i.e., 1 = very unimportant, 2 = unimportant, 3 = neutral, 4 = important, 5 = very important) and to provide the reasoning for their rating in separate surveys. Additionally, only the PE were questioned whether they agreed with the stated definition of each outcome. If they disagreed, an additional open-ended question was provided for an alternative definition. Both surveys included identical lay-language explanations for each outcome. The outcomes were grouped according to Dodds taxonomy and presented on six survey pages to aid overview.^13^

The survey responses were descriptively analyzed, and reasonings for outcome ratings were grouped according to similarities and themes, such as impact on life, expectation versus result, treatability, and occurrence rate. The study management group selected relevant quotes that illustrated these themes. To ensure neutrality, the study management group included quotes both supporting the importance of an outcome and those arguing against it whenever possible.

### Round 2

The second-round survey was the same for both stakeholder groups. Participants were shown all the outcomes from the first survey round on a single survey page. The outcomes were ranked in order based on their ratings from the first round, according to the combined means of LEE and PE, starting with the highest-rated outcome and numbered sequentially. Each outcome included a drop-down option that provided illustrative quotes from participants explaining the reasoning behind their ratings. Additionally, lay-language explanations for each outcome were still available. Participants were asked to indicate their level of agreement with the statement: “This outcome should be one of the ten outcomes that are always measured in research”. They could express their agreement on a 5-point Likert scale: 1 = strongly disagree, 2 = disagree, 3 = neither disagree nor agree, 4 = agree, 5 = strongly agree.

Descriptive statistics were used for analysis. Pre-defined consensus criteria (Table 1, section ‘Phase 2’) were applied to determine which outcomes met the inclusion or exclusion thresholds. Outcomes that met the inclusion criteria (i.e., at least 75% voting 4 or 5 and less than 15% scoring 1 or 2) from both stakeholder groups advanced to Phase 3, while those that met the exclusion criteria (i.e., 50% or fewer of both stakeholder groups scoring 4 or 5, or at least 75% scoring 1 or 2) were excluded from further consideration for the COS.

**Table 1:** Consensus criteria.

	Consensus classification	Description	Criteria
**Phase 2 (round 2 and 3)**			
5-point likert scale	Consensus in	Outcome recommended to be included in the Core Outcome Set	>75% scoring as 4 or higher AND <15% scoring 2 or less by both stakeholder groups
	Consensus out	Outcome to be excluded from the Core Outcome Set	50% or fewer scoring 4 or higher OR >75% scoring 2 or less by both stakeholder groups
	No consensus	Uncertainty regarding importance of outcome	Anything else
**Phase 3**			
5-point likert scale	Consensus in	Outcome recommended to be included in the Core Outcome Set	>70% of all participants scoring as 4 or 5
	Consensus out	Outcome to be excluded from the Core Outcome Set	50% or fewer of all participants scoring 4 or 5
	No consensus	Final decision to be made by the Study Steering Group	Anything else
Dichotomous vote	Consensus agree	Proposed statement accepted	>70% of all participants voting agree
	Consensus disagree	Proposed statement declined	>30% of all participants voting disagree

### Round 3

The third and final round only surveyed outcomes for which no consensus was reached. These outcomes were questioned in the same manner as in the second round, with the order based on the combined mean results from the second round. Outcomes that had received considerably different ratings between the LEE and PE groups (i.e., a difference of 10% or more between the percentages of LEE and PE rating an outcome as 4 or 5) during round two were marked with an additional note recommending that participants refer to the quotes for further insight.

At the end of the survey, participants were asked if they would like to take part in the consensus meeting and whether they would like to be acknowledged as contributors in the final publication. If they answered yes to either of the questions, they received a Google Form to provide their informed e-consent.

Responses were analyzed using descriptive statistics. Again, the pre-defined consensus criteria were applied to determine which outcomes were included and excluded. The outcomes that did not meet these criteria were categorized as follows; ‘borderline exclusion’ (outcomes that nearly met the exclusion criteria), ‘consensus on moderate importance’ (outcomes where the ratings of both groups were similar, but above the exclusion threshold and below the inclusion threshold), and ‘no consensus’ (in which only one stakeholder group's votes met the inclusion threshold, indicating a lack of agreement between the groups).

Before Phase 3, the study management group revised the definitions of the outcomes that were still being considered as potential core outcomes, using the feedback obtained from the first e-Delphi survey. Once the study steering group reviewed and approved these changes, the outcome definitions were finalized.

### Phase 3: finalizing the core outcome set

The goal was to host a consensus meeting including a total of 20 participants, ensuring equal representation from both stakeholder groups. To account for possible no-shows, approximately 25% more participants were invited. Purposeful sampling was employed to invite a diverse and balanced representation of stakeholders regarding healthcare profession (PE), years of experience (PE), (self-identified) gender identity, age, the type of gender-affirming surgical procedure undergone (LEE), time since surgery (LEE), and country of residence.

A preparatory guidance document was distributed to all participants prior to the meeting. In addition, an online question-and-answer session was organized beforehand so that participants could ask questions and familiarize themselves with Zoom Pro features.

The online consensus meeting was held using Zoom Pro software. It was conducted in English and moderated by an independent facilitator with experience in scientific consensus meetings. Participants were free to leave at any point during the meeting or to take extra breaks. The meeting was structured based on the results of the e-Delphi surveys. The outcomes in the categories ‘included’ and ‘excluded’ were briefly presented. The remainder of the meeting focused on the outcomes categorized as ‘borderline exclusion,’ ‘consensus on moderate importance,’ and ‘no consensus’, which were discussed in detail. In an effort to facilitate a balanced discussion and to help meeting attendees take into account the perspectives of participants from Phase 2, two measures were taken. First, each outcome was presented with detailed voting results from the final e-Delphi survey. Second, for outcomes in the ‘no consensus’ category, illustrative quotes collected in Phase 2, survey round 1 were additionally presented. Following the discussion of each outcome, participants voted on their agreement with statements proposing the inclusion or exclusion of an outcome (category) using a binary or 5-point Likert scale. The inclusion and exclusion criteria for outcomes based on the votes are outlined in Table 1, section ‘Phase 3.’ After each vote, the slide deck was updated in real-time to present the results. If consensus was not reached, the study steering group would discuss and decide later.

### Ethics

The interviews and focus group were part of a decision aid study that received ethical approval from the Amsterdam UMC, location VUmc ethical board (Reference numbers: 2020.0653 and 2021.0026), and the participants gave written consent for the data to be reused for ensuing research purposes. The GenderCOS Project received ethical approval from the Amsterdam UMC, location VUmc ethical board (Reference number: 2022.0102). Participants could only start with the e-Delphi surveys after providing an Informed e-consent.

### Role of the funding source

No funding was received for this study.

## Results

Fig. 1 provides an overview of the COS development stages and results.

### Protocol modifications

Minor amendments were made to the study protocol regarding Phase 2, and participants were informed of these between the e-Delphi survey rounds. The modifications were (i) Limesurvey survey software was used instead of Survalyzer software due to availability of software, (ii) the phrasing of the e-Delphi questions was adapted for better understanding, and (iii) the feedback initially planned for round two consisted of participants’ ratings alongside the median ratings of each stakeholder group. However, since most outcomes were rated highly, the feedback would likely have been too similar across outcomes, offering limited value for prioritization. To address this, the feedback was revised to present outcomes ranked by their mean ratings, making distinctions between outcomes more apparent and enhancing the prioritization process.

### Phase 1: identification of potential core outcomes

The systematic review identified 2077 individual outcomes, which were reduced to 384 unique outcomes. Eighteen interviews and one focus group with nine transgender men and gender diverse individuals were conducted. The qualitative data yielded 69 unique outcomes, all of which had also been identified in the systematic review.

Of the 384 unique outcomes, the study management group classified 91 as green or orange, and 293 as red. The study steering group agreed to exclude the outcomes classified as red and prioritized 38 out of the 91 as potential core outcomes. The formulated definitions and lay-language explanations for these 38 outcomes can be found in the Supplementary Material, Chapter 1.

### Phase 2: building consensus on outcome priority

#### e-Delphi participants

A detailed overview of the participant characteristics can be found in Table 2. Participants self-identified across a range of gender identities. Among the LEE, these included transgender men, non-binary, and agender individuals, while the PE identified primarily as cisgender men and women, with some identifying as genderqueer, transgender, or non-binary. Participants demonstrated geographic diversity, though most resided in the United States, the Netherlands, and the United Kingdom. Within the LEE group, participants had undergone various types of genital gender-affirming surgeries, with scrotoplasty and phalloplasty being the most common. Among the PE, there was a wide array of professional expertise, with over half being surgeons (i.e., plastic surgeons, urologists, gynecologists, and general surgeons), as well as a notable number of mental health professionals (i.e., psychiatrists, sexologists, and psychologists). Over half of the PE reported having more than 10 years of experience, with some exceeding 20 years.

**Table 2:** Demographics of study participants.

Demographic characteristic	Categories	Registrations	Phase 2, round 1	Phase 2, round 2	Phase 2, round 3	Phase 3
**Lived experience experts**						
Participation (response rate)		103	52 (50%)	35 (67%)	32 (91%)	2 (25%)
Gender-identity (self-identified)	Transgender man		36	24	23	1
	Non-binary		7	5	4	
	Genderqueer		0	0	0	
	Agender		2	1	1	
	Man		3	3	2	1
	Transsexual man		1	1	1	
	Trans man		3	1	1	
Country of residence	Argentina		2	1	1	
	Belgium		3	2	2	
	Canada		5	4	3	1
	The Netherlands		10	9	8	1
	Nicaragua		1	1	1	
	Norway		4	1	0	
	United Kingdom		5	3	3	
	United States of America		22	14	14	0
Age category	18–24 years old		4	2	2	
	25–34 years old		18	13	12	
	35–44 years old		13	10	8	1
	45–54 years old		8	5	5	
	55–64 years old		8	5	5	1
	65 years old and over		1	0	0	
Genital gender affirming surgical procedures (all that applied)	Phalloplasty with urethral lengthening		27	18	18	1
	Phalloplasty without urethral lengthening		10	4	3	
	Metoidioplasty with urethral lengthening		16	12	11	1
	Metoidioplasty without urethral lengthening		2	2	1	
	Scrotoplasty		35	25	24	2
	Coronaplasty		18	13	13	1
	*Total*		108	74	70	5
Time since first genital gender affirming surgery	3 months–1 year ago		5	3	3	
	1–2 years ago		8	5	5	
	3–5 years ago		13	8	7	
	6–9 years ago		13	9	7	1
	10–19 years ago		9	7	7	
	More than 20 years ago		4	3	3	1
**Professional experts**						
Participation (response rate)		228	66 (29%)	54 (82%)	52 (96%)	11 (65%)
Gender-identity (self-identified)	Transgender woman		2	2	2	1
	Non-binary		1	1	1	0
	Genderqueer		2	1	1	0
	Cisgender woman		17	12	11	1
	Cisgender man		37	32	31	9
	Transgender man		6	5	5	0
	Male (AFAB)		1	1	1	0
Country of residence	Argentina		1	1	1	1
	Belgium		4	3	3	0
	Canada		3	3	3	0
	France		1	1	1	0
	Germany		1	1	1	0
	Greece		1	1	1	1
	The Netherlands		15	15	15	3
	Sweden		2	1	1	0
	Switzerland		1	0	0	0
	Taiwan		1	1	1	0
	Thailand		1	1	1	0
	United Kingdom		10	9	9	3
	United States of America		25	17	15	3
Age category	18–24 years old		0	0	0	0
	25–34 years old		14	10	10	0
	35–44 years old		21	17	15	2
	45–54 years old		16	14	14	5
	55–64 years old		11	10	10	3
	65 years old and over		4	3	3	1
Current and/or past gender health care profession (all that apply)	Author of published research		29	24	24	7
	Endocrinologist		2	2	2	0
	General/abdominal surgeon		1	1	1	0
	Gynecologist		6	4	4	0
	Nurse		1	1	1	0
	Nurse Practitioner		1	0	0	0
	Physician Assistant		2	2	2	0
	Physiotherapist		2	1	1	0
	Plastic surgeon		20	18	17	5
	Psychiatrist		6	4	4	1
	Psychologist		7	7	7	2
	Sexologist		6	5	5	2
	Urologist		12	10	9	3
	*Other*					
	Anesthesiologist		1	1	1	1
	Author of national and international guidelines		1	1	1	1
	Care navigator specific to gender affirming surgery		1	0	0	0
	Conducted research		1	1	1	0
	Licensed Clinical and Social Worker Therapist		1	0	0	0
	Licensed Marriage and Family Therapist		1	1	1	0
	Occupational therapist		1	1	1	0
	Psychotherapist		1	1	1	1
	Registered Social Worker/Psychotherapist		1	1	1	0
	Research Professor		1	1	1	0
	Total		106	88	85	22
Duration of professional experience	2 years or less		7	4	3	0
	3–5 years		17	15	14	0
	6–9 years		15	10	10	4
	10–19 years		14	13	13	1
	20 years or more		13	12	12	6

AFAB, Assigned Female At Birth.

### Round 1

The first e-Delphi survey round opened February 2024. A total of 331 participants registered, of whom 17 did not meet the inclusion criteria and were excluded. Of those who did meet the eligibility criteria, 196 participants (156 PE, 40 LEE) started the survey but did not complete it, resulting in 118 participants who completed the first round.

Professional expert agreement with the proposed definitions for all 38 outcomes was high, with an average of 90.2%, ranging from 79.1% to 98.5% (see Table 3). The outcome *mean urinary flow rate* had the lowest agreement rate, along with general feedback that the outcome should be replaced by *maximum urinary flow rate*, arguing that this is a more relevant clinical outcome. Based on this, the study steering group changed the outcome in the following survey rounds.

**Table 3:** Summary of e-Delphi survey results.

	Outcome	Phase 2							Phase 3
		Round 1	Round 2			Round 3			
		PE agreement with definition (%)	Vote 4 or 5 (%)		Result	Vote 4 or 5 (%)		Result	Classification entering consensus meeting
			LEE	PE		LEE	PE		
1	Length of the neo-phallus	88.1	26.5	25.5	EX				
2	Tactile sensibility in the neo-phallus	83.6	70.6	70.9	NC	74.2	77.4	NC	Included
3	Flap necrosis of the neo-phallus	79.1	82.4	83.6	NC	80.6	84.9	IN	Included
4	Post-operative bleeding	95.5	11.8	40.0	EX				
5	Wound dehiscence	92.5	23.5	36.4	EX				
6	Delayed wound healing	91	20.6	34.5	EX				
7	Wound infection	85.1	26.5	49.1	EX				
8	Time until complete recovery	92.5	29.4	45.5	EX				
9	Need for re-intervention	83.6	61.8	63.6	NC	80.6	64.2	NC	No consensus
10	Readmission	95.5	23.5	38.2	EX				
11	Functional status of flap donor site	85.1	79.4	69.1	NC	67.7	58.5	NC	Consensus of moderate importance
12	Unplanned perineal urethrostomy	83.6	20.6	45.5	EX				
13	Ability to void in a standing position	80.6	67.6	67.3	NC	77.4	54.7	NC	No consensus
14	Neo-urethral stricture	91	73.5	70.9	NC	67.7	92.5	NC	No consensus
15	Neo-meatal stenosis	94	20.6	41.8	EX				
16	Neo-urethral fistula	91	85.3	69.1	NC	77.4	90.6	NC	Included
17	Post-void dribbling of urine	85.1	17.6	34.5	EX				
18	Post-void residual volume of urine	92.5	26.5	41.8	EX				
19	Mean urinary flow rate^a^	94	29.4	36.4	EX				
20	Genitals matching gender identity	88.1	52.9	56.4	NC	58.1	50.9	NC	Borderline exclusion
21	Feeling confident about genitals	92.5	47.1	32.7	EX				
22	Genital gender dysphoria	89.6	61.8	69.1	NC	61.3	64.2	NC	Consensus of moderate importance
23	Willingness to undergo genital gender surgery again	97	32.4	50.9	NC	45.2	43.4	EX	
24	Regret of decision to undergo genital gender surgery	86.6	32.4	50.9	NC	35.5	50.9	NC	Borderline exclusion
25	Regret of type of genital gender surgery undergone	95.5	26.5	43.6	EX				
26	Satisfaction with donor site aesthetic result	94	29.4	47.3	EX				
27	Acceptability of donor site morbidity	88.1	35.3	47.3	EX				
28	Satisfaction with neo-genital aesthetic result	98.5	91.2	81.8	IN				Included
29	Satisfaction with neo-phallus aesthetic result	98.5	52.9	50.9	NC	80.6	49.1	NC	Excluded
30	Surgical result matching expectations	94	47.1	58.2	NC	48.4	43.4	EX	
31	Satisfaction with surgical results	92.5	61.8	69.1	NC	54.8	67.9	NC	Consensus of moderate importance
32	Erogenous sensibility in neo-phallus	88.1	73.5	65.5	NC	77.4	64.2	NC	No consensus
33	Erogenous sensibility in the clitoral glans	86.6	29.4	41.8	EX				
34	Ability to perform sexual function as desired	88.1	52.9	65.5	NC	51.6	52.8	NC	Borderline exclusion
35	Ability to achieve orgasm	94	67.6	60.0	NC	74.2	47.2	NC	No consensus
36	Ability to perform penetrative sexual intercourse	86.6	26.5	43.6	EX				
37	Satisfaction with sexual function	92.5	64.7	63.6	NC	54.8	66.0	NC	No consensus
38	Overall sexual satisfaction	94	52.9	58.2	NC	67.7	56.6	NC	No consensus

PE, Professional Expert; LEE, Lived Experience Expert; EX, Excluded; NC, No Consensus; IN, Included.

a Mean urinary flow rate was changed to maximum urinary flow rate after Round 1.

The LEE and PE were asked to rate each outcome on importance. Both groups rated each outcome high with mean scores ranging from 3.16 to 4.57 on a 5-point Likert scale. A complete overview of the combined mean scores and of the quotes motivating participants’ outcome ratings can be found in Supplementary Material, Chapter 2A.

### Round 2

In May 2024, the second e-Delphi survey was opened to all participants who completed the first round. The second survey was completed by 89 participants, resulting in a total response rate of 75.4%. Of all 38 surveyed outcomes, only one outcome, satisfaction with neo-genital aesthetic result, met the criteria for recommended inclusion (see Table 1). Conversely, 18 outcomes met the exclusion criteria and were dropped altogether. Nineteen outcomes met neither the inclusion nor exclusion criteria and were therefore classified as ‘no consensus’. The combined mean ratings on the 5-point Likert scale of the outcomes in this category ranged from 3.19 to 4.26. Four outcomes were rated considerably different between the PE and LEE groups (i.e., a 10% points or more difference between the percentages of PE and LEE that rated an outcome as 4 or 5). These were the *functional status of the flap donor site, neo-urethral fistula, ability to perform the sexual function as desired,* and *regret of the decision to undergo genital gender surgery*. Summarized voting results of the second survey round are presented in Table 3, and detailed ranking results can be found in Supplementary Material, Chapter 2B and 2C.

### Round 3

The third and final e-Delphi survey was held in July 2024. The response rate was 94.4%, with 84 participants who completed the survey. Of the 19 surveyed outcomes, one (*flap necrosis of the neo-phallus*) met the criteria for recommended inclusion (see Table 1, section ‘Phase 3’). Two met the exclusion criteria, and sixteen reached no consensus (see Table 3 for the summarized results).

In preparation for Phase 3, the study steering group reviewed the results of Round 3 and categorized the outcomes accordingly (see Table 3, and Supplementary Material, Chapter 2C). Two outcomes—*tactile sensibility in the neo-phallus* and *neo-urethral fistula*—came close (i.e., within 1.2%) to meeting the recommended inclusion criteria. The study steering group opted to classify these as included to streamline the consensus meeting and prioritize discussions on more contentious outcomes. Due to the similarity of the outcomes *satisfaction with sexual function* and *overall sexual satisfaction*, the study steering group decided to propose that they be merged into *sexual well-being* and classified as ‘no consensus’ for further discussion in Phase 3.

### Phase 3: finalizing the core outcome set

The consensus meeting was organized in September 2024. A total of 32 (8 LEE, 24 PE) e-Delphi participants showed interest in participating. Eight LEE and seventeen PE were invited to participate, of whom two LEE and eleven PE were present at the meeting. The demographics of the consensus meeting participants are described in Table 2, column ‘Phase 3’. The consensus meeting lasted 3 h.

A total of 10 votes were held during the meeting. The details of the votes, the number of participants who voted, and the results can be found in Table 4. The outcome *functional status of flap donor site* was initially categorized as ‘consensus on moderate importance’. During the discussion of this outcome category, the participants expressed the wish to change this outcome to *donor site morbidity* and to discuss and vote on as a separate outcome (vote 3). The vote for the category ‘consensus on moderate importance’ was continued with the two remaining outcomes in this category (vote 2). Throughout the meeting, consensus was reached on the inclusion or exclusion of all previously undecided outcomes.

**Table 4:** Summary of consensus meeting.

Number	Vote (type of vote)	Type of vote	Outcome category	Outcome(s)	Total number of voters	Vote result	Result
1	‘The outcomes in the category borderline exclusion should not be included in the Core Outcome Set’	Yes/no	Borderline exclusion	Ability to perform sexual function as desired, genitals matching gender identity and regret of decision to undergo genital gender surgery.	10	90% agree	Excluded
2	The outcomes in the category consensus moderate importance should not be included in the Core Outcome Set'	Yes/no	Consensus of moderate importance	Satisfaction with surgical results and genital gender dysphoria.	9	100% agree	Excluded
3	‘How much do you disagree or agree that this outcome should be included in the Core Outcome Set?’	5-point Likert scale	Consensus of moderate importance	Donor site morbidity	8	88% agree or strongly agree	Included
4	‘How much do you disagree or agree that this outcome should be included in the Core Outcome Set?’	5-point Likert scale	No consensus	Neo-urethral stricture	8	100% agree or strongly agree	Included
5	‘How much do you disagree or agree that this outcome should be included in the Core Outcome Set?’	5-point Likert scale	No consensus	Erogenous sensibility in neo-phallus	8	88% agree or strongly agree	Included
6	‘How much do you disagree or agree that this outcome should be included in the Core Outcome Set?’	5-point Likert scale	No consensus	Need for additional surgery	8	75% agree or strongly agree	Included
7	‘How much do you disagree or agree that this outcome should be included in the Core Outcome Set?’	5-point Likert scale	No consensus	Ability to void in a standing position	9	100% agree or strongly agree	Included
8	‘How much do you disagree or agree that this outcome should be included in the Core Outcome Set?’	5-point Likert scale	No consensus	Ability to achieve orgasm	8	76% agree or strongly agree	Included
9	‘These outcomes should be merged into a single outcome: “Sexual well-being”.’	Yes/no	No consensus	Satisfaction with sexual function and overall sexual satisfaction	8	100% agree	Merged
10	‘How much do you disagree or agree that this outcome should be included in the Core Outcome Set?’	5-point Likert scale	No consensus	Sexual well-being	7	100% agree or strongly agree	Included

During the consensus meeting, it was suggested to merge the outcomes *erogenous sensibility in the neo-phallus* and *tactile sensibility in the neo-phallus* into a single outcome *sensibility in the neo-phallus*. Following this meeting, the study steering group implemented the suggestion and modified the outcome definition accordingly. Furthermore, the outcome *need for additional surgery* was changed to *additional surgery*.

### Final COS for masculinizing gGAS

The final COS for masculinizing gGAS is presented in Table 5. The COS is organized in a modular format with the first five outcomes being relevant to all masculinizing gGAS, while other outcomes apply specifically to urethral lengthening and phalloplasty. Table 5 includes definitions for the outcomes within the COS.

**Table 5:** Core outcome set for masculinizing genital gender-affirming surgery.

Scope	#	Outcome	Type of outcome	Definition	Lay language explanation
Masculinizing genital gender affirming surgery (all)	1	Sensibility in the neo-phallus	Patient reported outcome	Degree of experienced sensation (i.e., pressure, touch, sexual, temperature) in the surgically constructed penis.	How well someone can feel sensations—such as touch, pressure, temperature, or sexual stimulation—in their surgically created penis.
	2	Additional surgery	Clinical outcome	Additional surgical intervention needed to deal with complications, suboptimal outcomes, perceived problems or non-achieved desired results following previous genital gender affirming surgery.	The need for another surgery after genital gender-affirming surgery, because of complications, problems, or results that didn't meet expectations.
	3	Ability to achieve orgasm	Patient reported outcome	Ability to experience a sexual climax	The ability to experience a sexual climax.
	4	Sexual well-being	Patient reported outcome	A person's state of physical, emotional, mental and social well-being in relation to sexuality.	How someone feels physically, emotionally, mentally, and socially in relation to their sexuality.
	5	Satisfaction with neo-genital aesthetic result	Patient reported outcome	Satisfaction with the aesthetic result of the surgically created phallus and, if applicable, scrotum.	How satisfied someone is with how their surgically created genitals (penis and, if applicable, scrotum) look.
Urethral lengthening	6	Neo-urethral fistula	Adverse event	Unnatural connection between the surgically created urethra and another adjacent open space within or outside the body.	An abnormal connection between the surgically created urethra and another nearby area inside or outside the body.
	7	Neo-urethral stricture	Adverse event	Narrowed segment of the surgically constructed urethra due to fibrosis and cicatrization of the urethra, associated with obstructive voiding symptoms.	A narrowing of the surgically created urethra caused by scarring, which can block urine flow and lead to problems like a weak stream or difficulty urinating.
	8	Ability to void in a standing position	Patient reported outcome	Ability to urinate while standing with a directable stream, through an unzipped fly without removing lower body clothing, without a urination device, allowing the use of public restrooms.	The ability to pee while standing up with a steady stream, without needing a special device or having to remove clothing—allowing someone to use a public toilet like a urinal.
Phalloplasty	9	Flap necrosis of the neo-phallus	Adverse event	Portion of the flap, used to surgically construct the phallus, that has died.	Whether and how much of the skin or tissue used to surgically create the penis dies after surgery.
	10	Donor site morbidity	Patient reported outcome	Degree of experienced physical adverse effects (e.g., pain, sensation, tightness, discomfort, pulling) on the flap donor site.	How the area of the body where skin or tissue was taken to create the penis is affected after surgery — for example, through pain, tightness, discomfort, pulling, or changes in sensation.

## Discussion

In this study a COS for all research into masculinizing gGAS was developed to address the current lack of standardization and consistency in outcome reporting. Through a multi-phase process, international key stakeholders agreed on the COS for masculinizing gGAS. Depending on the type of procedure, the final modular COS consists of up to 10 outcomes. These outcomes should, at a minimum, be measured and reported in all future research into masculinizing gGAS.

Our systematic review identified 384 unique outcomes previously reported in masculinizing gGAS research. Of the ten most frequently reported outcomes in the literature, six are included in the COS, either verbatim or in a related form; *neo-urethral fistula, neo-urethral stricture, the ability to void in a standing position, flap necrosis of the neo-phallus, sensibility in the neo-phallus,* and *additional surgery*. While there is some overlap between the COS and the most frequently reported outcomes, our findings highlight that commonly reported outcomes may not always align with those considered most important to stakeholders. Notably, six of the ten included core outcomes are patient-reported outcomes (PROs), whereas only two of the ten most reported outcomes in the literature are PROs.^3^ This difference likely reflects the involvement of LEE perspectives in this study, supporting the need for including LEE voices in masculinizing gGAS research (design).

The majority of outcomes reported in research on masculinizing gGAS are adverse events or additional interventions.^4^^,^^14^ However, these are often ill-defined and inconsistently assessed.^3^ Although the inclusion of specific adverse events in a COS has generally been approached with caution in the literature,^15^ this study chose to include them for several reasons: (i) adverse events were frequently highlighted as important by trans and gender diverse individuals in the interviews and focus group conducted during phase 1; (ii) due to the high complication rates associated with masculinizing gGAS, they are a substantial aspect of this care, (iii) to improve the reporting and standardization of adverse events, and (iv) insight into adverse events is critical for informed surgical decision-making. While some COS developers group all adverse events under a single outcome domain labeled “adverse events”,^16^ this study determined that such an approach would lack the necessary granularity to capture the wide range of complications. Instead, this COS includes the most meaningful adverse events to enhance their reporting, facilitating key outcome data that differentiates one surgical approach from another (e.g., urinary issues in surgery with or without urethral lengthening).

A notable observation was the contrasting views of LEE and PE on certain adverse events in the first e-Delphi survey round. PE often focus on the treatability of these events, emphasizing how clinically manageable they are. Conversely, LEE highlighted the impact these events and their management had on daily lives and overall well-being. These varying perspectives between LEE and PE could be valuable in shared decision-making regarding surgical treatment and demonstrated the added value of the inclusion of both stakeholder groups in this study. Additionally, within the LEE group, participants may have had differing perspectives based on their individual experiences with surgical treatments, particularly regarding any specific adverse events they may have encountered. This diversity in personal experiences likely influenced how participants prioritized outcomes. Diverse prioritization could have resulted in less consensus and only identifying the mutual outcomes. However, the inclusion of core outcomes specific to certain masculinizing gGAS procedures indicates that LEE participants also considered what would be important to the stakeholder group beyond their own experiences. The COS’ modular design represents multiple perspectives and accommodates various surgical treatments.

The final COS includes aesthetic, sexual, functional and adverse event outcomes. The aesthetic, sexual, and functional outcomes reflect specific goals that transgender and gender-diverse individuals may have when considering masculinizing gGAS. These outcomes are specific enough to compare different procedures in light of these motivations and evaluate the effectiveness of each procedure. The adverse events included in the COS highlight the need for a comprehensive understanding of their risks. These outcomes are fundamental for assessing the safety of surgical procedures. The COS facilitates balancing personal surgical goals with the effectiveness and safety of various masculinizing gGAS procedures, contributing directly to informed, evidence-based surgical decision-making.

Methodological initiatives have defined standards for development and reporting of COSs.^6^^,^^9^^,^^17^ While the current study has followed these standards and recommendations, several methodological findings and aspects warrant discussion (see also Supplementary Material, Chapter 3). This study aimed to conduct three rounds of e-Delphi surveys to build consensus on the importance of including outcomes in the COS. However, it could be argued that only two rounds were conducted effectively, as all outcomes received uniformly high ratings in round one. This immediate consensus was likely the result of the phrasing of the questions and the Likert scale labels, since none of the participants considered any of the 38 outcomes “very unimportant (1)” or “unimportant (2).” The wording of the questions and scale was revised in the second and third rounds to provide more nuance in rating, clarify the study's goal, and improve understanding. These changes resulted in a more diverse range of voting and prioritization of outcomes, demonstrating the efficacy of the survey rephrasing.

Furthermore, the methodology lacks guidance on handling outcomes on which consensus is reached as being of moderate importance: they fall under the category ‘no consensus’,^6^ even though there is agreement that they are relevant but not crucial for inclusion in a COS. In this study it was determined that outcomes where one stakeholder group's rating met the inclusion criteria, while the other stakeholder group's rating did not, should be classified as ‘no consensus’. During the consensus meeting, it was proposed to exclude outcomes categorized as ‘consensus on moderate importance,’ as no stakeholder group considered them essential for inclusion in the COS. Future COS developers should consider this methodological gap when defining the consensus criteria.

This study has several strengths. It employed rigorous scientific consensus methods, particularly through three e-Delphi surveys and a consensus meeting. This COS meets the methodological standards established by the COMET Initiative.^6^^,^^9^^,^^17^ LEE were involved at every stage of the development process. The study steering group included LEE and PE from various geographic regions, representing a diverse range of healthcare professions and systems. The study protocol was published before the first e-Delphi survey began to ensure transparency.^7^ During phase two (i.e., three rounds of e-Delphi surveys) of the study, a diverse demographic representation was achieved. The e-Delphi surveys were provided in multiple languages to maximize global representation.

The strengths of this study are balanced with some limitations. It should be recognized that there are subjective components to the consensus process, particularly in determining which outcomes were taken forward to phase 2 and which quotes were selected for presentation in e-Delphi rounds two and three. Despite efforts to recruit globally, participants in Europe, the UK, and the USA responded strongly. Conversely, stakeholders in Asia, Africa, and South America were limited in representation. This imbalance largely reflects the origins of masculinizing gGAS research and the current focus of COS implementation. To ensure the COS remains relevant across diverse global contexts, future evaluation and implementation efforts should intensify efforts to actively incorporate perspectives from underrepresented regions. The online Delphi methodology may have created barriers for people with lower language and digital skills. Furthermore, many registered participants failed to start or complete the first e-Delphi survey. The extensive first round survey, which included 38 outcomes to be assessed twice (LEE) and threefold (PE), may have been a contributing factor to the high rate of survey non-completion. The low attrition rate (5.6%) between the second and third survey rounds makes possible attrition bias unlikely in this phase of the study (i.e., survey rounds in which outcomes were included or dropped). Further research is needed to determine the optimal survey length in COS development to maintain participant engagement without introducing bias. This study aimed to invite equal representation for each stakeholder group to the consensus meeting, but the meeting resulted in a low attendance from the LEE group. The LEE group rated six out of seven ‘no consensus’ outcomes higher in the e-Delphi surveys. However, during the consensus meeting, the PE group shifted closer to the LEE group's viewpoints, and through the discussions held, all six outcomes were ultimately included in the COS.

This COS represents a minimum set of outcomes that should be measured and reported in all clinical research into masculinizing gGAS and is an essential step towards standardization of research. It is important to emphasize that researchers may include additional outcomes to those in the COS when relevant to their study. The COS should be subject to ongoing evaluation to ensure its relevance. The final COS can potentially improve data harmonization in masculinizing gGAS research, enabling robust evidence-based surgical treatment decision-making and guideline development. Ultimately, this can improve the quality of masculinizing gGAS care. Future steps within the GenderCOS project include achieving consensus on how and when the core outcomes should be measured. These upcoming steps will continue to incorporate global stakeholder perspectives.

## Contributors

Conceptualization: all authors contributed equally. Data curation: PJR, MSV, MA. Formal analysis: PJR, MSV, MGM. Methodology: PJR, MSV, TEP, MGM. Project administration: PJR, MSV. Resources: all authors contributed equally. Software: PJR, MSV. Supervision: MGM. Translations: JB, MB, AC, PJR, MSV. Writing–original draft: PJR. Writing–review & editing: all authors contributed equally. All authors read and approved the final version of this manuscript. PJR, MSV, MA and MGM had direct access to and verified the underlying data.

## Data sharing statement

The data supporting this study's findings are primarily available in the supplemental material. Any additional data are available upon reasonable request from the corresponding author.

## Declaration of interests

Author WPB has served as a paid consultant for Karo Healthcare (Stockholm, Sweden), as an unpaid President of the WPATH (past), and as a paid Editor-in-Chief of the *International Journal of Transgender Health* (past). All other authors declare no conflicts of interest.

## References

[1] Chaya B.F., Berman Z.P., Boczar D. (2022). Gender affirmation surgery on the rise: analysis of trends and outcomes. LGBT Health.

[2] Al-Tamimi M., Pigot G.L., Elfering L. (2020). Genital gender-affirming surgery in transgender men in the Netherlands from 1989 to 2018: the evolution of surgical care. Plast Reconstr Surg.

[3] Roijer P.J., Vallinga M.S., Jorna M.M.F. (2024). Navigating toward standardization: a systematic review mapping outcome measures in masculinizing genital gender affirming surgery. Int J Transgend Health.

[4] Wang A.M.Q., Tsang V., Mankowski P., Demsey D., Kavanagh A., Genoway K. (2022). Outcomes following gender affirming phalloplasty: a systematic review and meta-analysis. Sex Med Rev.

[5] Butcher R.L., Kinney L.M., Blasdel G.P. (2023). Decision making in metoidioplasty and phalloplasty gender-affirming surgery: a mixed methods study. J Sex Med.

[6] Williamson P.R., Altman D.G., Bagley H. (2017). The COMET handbook: version 1.0. Trials.

[7] Roijer P.J., Vallinga M.S., Pidgeon T.E. (2023). The GenderCOS project: study protocol for the development of two international core outcome sets for genital gender affirming surgery. Int J Transgend Health.

[8] Core Outcome Measures in Effectiveness Trials (COMET) database: registration study number 2067. https://www.comet-initiative.org/Studies/Details/2067.

[9] Kirkham J.J., Gorst S., Altman D.G. (2019). Core outcome set-STAndardised protocol items: the COS-STAP statement. Trials.

[10] Roijer P.J., Vallinga M.S., Mullender M.G., Pidgeon T., Bouman M., Ket J.D.F. (2022). https://www.crd.york.ac.uk/prospero/display_record.php?ID=CRD42022347400.

[11] Mokken S.E., MDvE The GenderAID: a decision aid for genital gender surgery. https://genderaid.org/en.

[12] McMillan S.S., King M., Tully M.P. (2016). How to use the nominal group and Delphi techniques. Int J Clin Pharm.

[13] Dodd S., Clarke M., Becker L., Mavergames C., Fish R., Williamson P.R. (2018). A taxonomy has been developed for outcomes in medical research to help improve knowledge discovery. J Clin Epidemiol.

[14] Hu C.H., Chang C.J., Wang S.W., Chang K.V. (2022). A systematic review and meta-analysis of urethral complications and outcomes in transgender men. J Plast Reconstr Aesthet Surg.

[15] Tay J., Robinson C., Blazeby J. (2024). Inclusion of harm outcomes in core outcome sets requires careful consideration. J Clin Epidemiol.

[16] COMET Initiative (2018). Outcome classification: taxonomy: explanation and examples. https://www.comet-initiative.org/Resources/OutcomeClassification.

[17] Kirkham J.J., Gorst S., Altman D.G. (2016). Core outcome set-STAndards for reporting: the COS-STAR statement. PLoS Med.

[18] LTD MMS (2024). The medical moderator. https://themedicalmoderator.com/.

