# Supplementary material for: A core outcome set for research on masculinizing genital gender-affirming surgery: international consensus results from the GenderCOS project
Source: eClinicalMedicine. 2025 Jul 1;85:103325. doi: 10.1016/j.eclinm.2025.103325 (PMC12308309; doi:10.1016/j.eclinm.2025.103325)
Supplement: Supplementary material [file mmc1.pdf]

Supplementary material - A Core Outcome Set for Research on Masculinizing Genital Gender-Affirming Surgery: International Consensus Results from The GenderCOS Project

Table of contents – Supplementary material

1. Results Phase 1: Identification of potential core outcomes 2
2. Results Phase 2: Building consensus on outcome priority
   A. Round 1 6
   B. Round 2 13
   C. Round 3 15
3. Discussion 17

Chapter 1
Results Phase 1: Identification of potential core outcomes

Table 1. List of 38 identified potential core outcomes.

	Masculinizing genital gender-affirming outcome	Definition	Lay language explanation
1	Length of the neo-phallus	Length of the surgically constructed penis in centimetres, from the tip of penis to the pubic bone, flaccid and un-stretched, with the implant deflated if present, as assessed by a clinician. Time of outcome assessment must be reported.	Length of the surgically created penis, measured in centimetres from the tip of the penis to where it attaches to the body.
2	Tactile sensibility in the neo-phallus	Ability to perceive and interpret the sensation of touch on the skin of the surgically constructed penis, as assessed clinically. Time of outcome assessment must be reported.	How well someone can feel it when the skin of their surgically created penis is touched.
3	Flap necrosis of the neo-phallus	Proportion (as an estimated percentage) of the skin flap, used to surgically construct the penis, that has died due to ischaemia, as assessed by a clinician. Outcome must be graded according to the Clavien-Dindo classification. Time of outcome assessment must be reported.	Whether and how much of the skin or tissue used to create the penis has died after surgery. In some cases, some or all of this skin may die. Depending on the severity, surgery may be needed. The size and shape of the penis may also be affected.
4	Post-operative bleeding	Bleeding at the surgical site after leaving the operating theatre, as assessed clinically and/or using imaging. Outcome must be graded according to the Clavien-Dindo classification. Time of outcome assessment must be reported.	More bleeding after the surgery than what is expected to be normal for this procedure. This could mean that a blood vessel in the surgical wound does not stop bleeding on its own. Depending on the severity, management may involve applying pressure, a blood transfusion or surgery.
5	Wound dehiscence	Separation of the edges of surgical incisions or wounds that were primarily closed during surgery, as assessed by a clinician. Outcome must be graded according to the Clavien-Dindo classification. Time of outcome assessment must be reported.	The opening of a wound that has been closed after the surgery. Sometimes the stiches or bandages don't hold the edges of the surgical wound together well enough. This could be due to a lot of tension on the wound, for example due to (excess) movement. This can result in a higher risk of infection of the wound, as there is a larger wound area exposed. It can also take more time for the wound to heal.
6	Delayed wound healing	Failure of surgical incisions or wounds (skin grafts excluded) that were primarily closed during surgery to heal to a stable scar within 3 weeks, as assessed by a clinician. Outcome must be graded according to the Clavien-Dindo classification. Time of outcome assessment must be reported.	After an average surgery, it can be expected for a surgical wound to take around 3 weeks to heal. Bigger surgeries may take longer and smaller surgeries may take shorter. Delay in the expected time of healing can be due to multiple factors, such as wound infection or wound dehiscence.
7	Wound infection	Clinical signs of surgical site infection, as assessed by a clinician with or without a change in management. Outcome must be graded according to the Clavien-Dindo classification. Time of outcome assessment must be reported.	When a bacteria or other germs get inside the surgical wound. This can cause the area of the wound to become red, swollen and painful. A progressed infection can also lead to fever and becoming ill. Depending on the severity, management may involve cleaning of the surgical wound, medication (such as antibiotics) or surgery.
8	Time until complete recovery	Time taken to fully heal and return to their normal level of activity and pre-operative physical state, as reported by the patient. Time of outcome assessment must be reported.	The time period it takes for someone's body to heal after surgery and return to its normal or before-surgical physical state. This entails returning to usual activities, free from surgically induced limitations. This period varies depending on how big the surgery is.
9	Need for re-intervention	Any medical or surgical intervention needed to deal with a complication or problem related to the previous genital gender affirming surgery. Time of outcome assessment must be reported.	When someone needs additional medical treatment or surgery to deal with a complication or problem related to their previous surgery.

10	Readmission	Unplanned hospital readmission after discharge, due to complications or problems related to the previous genital gender affirming surgery. Time of outcome assessment must be reported.	When someone needs to be readmitted to hospital to receive care and medical attention after being discharged from the hospital following their previous surgery.
11	Unplanned perineal urethrostomy	Perineal urethrostomy as a part of the management of a complication or problem related to the previous genital gender affirming surgery. Time of outcome assessment must be reported.	An unplanned surgically created opening in the area between the anus and the surgically created scrotum (the perineum), through which someone can pee. This may be indicated when someone cannot pee through the intended route.
12	Ability to void in a standing position	Ability to urinate while standing with a directable stream, without leakage or spraying of urine, as reported by the patient and/or assessed by a clinician. Time of outcome assessment must be reported.	The ability to pee while standing with a directable stream, without leakage or spraying of urine.
13	Neo-urethral stricture	Narrowing of the surgically constructed urethra, resulting in lower urinary tract symptoms, as assessed clinically and/or using imaging and/or endoscopy. Outcome must be graded according to the Clavien-Dindo classification. Time of outcome assessment must be reported.	A narrowing in the surgically created urethra, which is the tube that carries urine from the bladder to the outside of the body. This can obstruct the urine, leading to difficulties like prolonged peeing time, a weak stream, or, in severe cases, inability to pee. Depending on the severity, management may involve widening or surgery.
14	Neo-meatal stenosis	Narrowing of the surgically constructed urethral opening, resulting in lower urinary tract symptoms, as assessed by a clinician. Outcome must be graded according to the Clavien-Dindo classification. Time of outcome assessment must be reported.	A narrowing of the opening of the surgically created urethra, which is where the urine leaves the body. This can obstruct the urine, possibly leading to difficulties like a prolonged peeing time, a weak stream, or, in severe cases, an inability to pee. Depending on the severity, management may involve widening or surgery.
15	Neo-urethral fistula	Abnormal connection between the surgically constructed urethra and another adjacent structure or area in or outside of the body, as assessed clinically and/or using imaging or endoscopy. Outcome must be graded according to the Clavien-Dindo classification. Time of outcome assessment must be reported.	An abnormal passageway that has developed between the surgically created urethra and another nearby area or organ. This can lead to urine leakage into unintended areas in- or outside of the body. Depending on the severity, management may involve surgery.
16	Post-void dribbling of urine	Involuntary loss of urine that occurs after a completed act of urination, as reported by the patient and/or assessed by a clinician. Outcome must be graded according to the Clavien-Dindo classification. Time of outcome assessment must be reported.	Involuntary loss of urine after peeing. This can happen after someone believes they have finished peeing. Depending on the severity and amount of urine involuntary lost, management may involve wearing absorbent protection, urethral milking or surgery.
17	Post-void residual volume of urine	Volume of urine remaining in the bladder after a completed act of urination in millilitres, as assessed by a clinician using catheter drainage and/or imaging. Time of outcome assessment must be reported.	The amount of urine that remains in the bladder after someone has emptied it by peeing to the best of their ability. When this is more than expected, it may indicate a decreased urinary function. Measuring the residual urinary volume can help to monitor urinary function and diagnose urinary problems.
18	Mean urinary flow rate	Mean rate of urine flow during urination in millilitres per second, as assessed clinically using uroflowmetry. Time of outcome assessment must be reported.	The average speed at which urine leaves the body while peeing. This provides information about someone's urinary function. A low average flow rate, meaning that the stream is weak and it takes a longer time than expected to urinate, may indicate a decreased urinary function. Measuring the average urinary flow rate can help monitoring urinary function and diagnosing urinary problems.
19	Functional status of flap donor site	Ability of the donor site to return to the expected level of activity and physical function, as reported by the patient. Time of outcome assessment must be reported.	The ability of the donor site to function in a similar way to before surgery. The donor site is the area of the body from which tissue and/or skin was taken to make the penis. This measure helps to determine the effect of the surgery on the ability to move or function in daily life.
20	Genitals matching gender identity	Extent to which the genitals match the gender identity, as reported by the patient. Time of outcome assessment must be reported.	The extent to which someone feels that their genitals match their gender identity.
21	Feeling confident about genitals	Feeling of confidence about their genitals, as reported by the patient. Time of outcome assessment must be reported.	The extent to which someone feels confident about their genitals.
22	Genital gender dysphoria	Distress and/or discomfort related to the incongruence between the gender identity and the physical genitals, as reported by the patient. Time of outcome assessment must be reported.	Experience of distress and/or discomfort related to the mismatch between someone's gender identity and the physical appearance of their genitals.

23	Willingness to undergo genital gender surgery again	Whether, with the benefit of hindsight, the same surgical treatment would be chosen again, as reported by the patient. Time of outcome assessment must be reported.	Whether, looking back, someone would choose to undergo the same genital gender affirming surgery again based on their experiences.
24	Regret of decision to undergo genital gender surgery	Regret relating to having undergone genital gender affirming surgery, as reported by the patient. Time of outcome assessment must be reported.	Feeling of regret having undergone genital gender affirming surgery. This indicates that someone regrets their decision to undergo surgery, regardless of the type of surgery performed.
25	Regret of type of genital gender surgery undergone	Regret about the type of undergone genital gender affirming surgery, as reported by the patient. Time of outcome assessment must be reported.	Feelings of regret about choosing a particular type of genital gender affirming surgery, but having no regret for choosing to undergo genital gender affirming surgery in general.
26	Satisfaction with donor site aesthetic result	Satisfaction with the aesthetic result of the completely healed donor site, as reported by the patient. Time of outcome assessment must be reported.	Level of satisfaction with the physical appearance of the completely healed donor site. The donor site is the area of the body from which tissue and/or skin was taken to create the penis.
27	Acceptability of donor site morbidity	Acceptance of donor site morbidity in the context of the surgical goal for which the tissue was donated, as reported by the patient. Time of outcome assessment must be reported.	Level of tolerance for the physical effects on the area of the body from which tissue and/or skin was taken to create the penis, in context of the goal of that surgery.
28	Satisfaction with neo-genital aesthetic result	Satisfaction with the aesthetic result of the surgically created external genitals, as reported by the patient. Time of outcome assessment must be reported.	Level of satisfaction with the physical appearance of the surgically created external genitals. The external genitals include the penis and scrotum.
29	Satisfaction with neo-phallus aesthetic result	Satisfaction with the aesthetic result of the surgically created phallus, as reported by the patient. Time of outcome assessment must be reported.	Level of satisfaction with the physical appearance of the surgically created penis.
30	Surgical result matching expectations	The extent to which the surgical result matches the pre-surgical expectations, as reported by the patient. Time of outcome assessment must be reported.	The extent to which the result of the genital gender affirming surgery matches someone's expectations before the surgery.
31	Satisfaction with surgical results	Satisfaction with the surgical result, as reported by the patient. Time of outcome assessment must be reported.	Level of satisfaction with the surgical result.
32	Erogenous sensibility in neo-phallus	Ability to perceive erogenous sensation in the surgically created phallus, as reported by the patient. Time of outcome assessment must be reported.	The ability to experience sexual sensations in the surgically created penis.
33	Erogenous sensibility in the clitoral glans	Ability to perceive erogenous sensation in the clitoral glans, as reported by the patient. Time of outcome assessment must be reported.	The ability to experience sexual sensations in the clitoral glans. The clitoral glans is the anatomical description of the head of the clitoris. The location of the clitoral glans can vary, depending on the type of surgery someone has received.
34	Ability to perform sexual function as desired	Ability to function sexually with their genitals as desired, as reported by the patient. Time of outcome assessment must be reported.	Ability to use genitals as someone desires during sexual activity. This assesses someone's ability, but not how their genitals are used exactly. Examples may include: the ability to have and hold an erection, achieving an orgasm, and being able to penetrate during sexual activity.
35	Ability to achieve orgasm	Ability to experience a sexual climax, as reported by the patient. Time of outcome assessment must be reported.	Ability to experience a sexual climax. This assesses someone's ability, but not how it is experienced or reached.
36	Ability to perform penetrative sexual intercourse	Ability to perform penetrative sexual intercourse, as reported by the patient. Time of outcome assessment must be reported.	Ability to use the genitals to penetrate another partner's preferred body opening. This may include the vagina, anus and/or mouth.
37	Satisfaction with sexual function	Satisfaction with the way the genitals function in relation to the sexual needs, as reported by the patient. Time of outcome assessment must be reported.	Level of satisfaction with the way someone's genitals function in relation to their sexual needs. This assesses someone's level of satisfaction with function, but not what these functions are exactly. Examples may include: the ability to have and hold an erection, achieving an orgasm, and being able to penetrate during sexual activity.

38	Overall sexual satisfaction	Overall sexual satisfaction, as reported by the patient. Time of outcome assessment must be reported.	Experienced level of overall sexual satisfaction. This encompasses various sexual aspects that may include: sexual health, intimacy, desire, arousal, emotional connections, communication, sexual functions, relationship satisfaction and trust.

Chapter 2A

Results Phase 2: Building consensus on outcome priority - Round 1

Table 2. Results of Phase 2: e-Delphi survey round 1

Survey round 1 order	Outcome	Mean of combined LEE & PE ratings (SD)	Median of combined LEE & PE ratings (Q1-Q3)	Illustrative quotes
1	Length of the neo-phallus	3·75 (1·05)	4 (3-4)	"For me, length is important for both sex, being able to void while standing and for alignment. But it's about functional length, not maximum length for me, so measurement is less important."<br>"I understand that this cannot be controlled and depends on many individual factors. I would be interested to know what the safe range would be, where length corresponds with the lowest rate of complications. I think that would be valuable information when deciding on surgery."<br>"It changes over time, it also shrinks with every re-intervention, I wasn't prepared for that in advance. The shape changed as well. I wonder if other people experience changes over time as well."<br>"In our society, penile length is associated with masculinity, therefor, many patients wonder about the length before surgery. They can experience dysphoria and/or euphoria depending on the outcome. Personally I don't think it should matter, but I know patients find this important."
2	Tactile sensibility in the neo-phallus	4·43 (0·77)	5 (4-5)	"I wanted to feel a sense of connection to my penis. Without feeling it, it doesn't feel a part of you and can be dysphoria triggering. Feeling that it's touched is validating and comforting for safety as well."<br>"This is really important. I wanted feedback when being intimate. I need sensation for sexual pleasure. But before surgery, the info on it was really vague. That was difficult for me, because I didn't really know what to expect. More research would be important."<br>"I don't think it's the be-all, end-all outcome, because it is also very subjective. It would be important to know whether the people who don't have sensation are bothered by it. I had sensation in the beginning, but due to complications it declined. I'm not that bothered."<br>"It's an important outcome for many functions; body alignment/integration, sex, urinating, proprioception, protection and safety. But it is also difficult to measure objectively, how to measure this in a reliable way is subject for discussion."
3	Flap necrosis of the neo-phallus	4·45 (0·79)	5 (4-5)	"I did not have any necrosis and was extremely grateful for that. For me it was the most feared complication. The whole point of surgery is to have a penis and it dying would be horrible. More data on this would help make informed decisions."<br>"I chose a technique where the chance of this happening was a bit smaller. But I think it should be researched more, the presence of necrosis to any degree, alongside flap choice, size, placement and other surgical details. Flap selection should be based on risk management."<br>"When severe, it can have huge impact on the size and function of the phallus. For example on being able to stand while peeing, or complicating the insertion of an erectile prosthesis. This can have consequences for the overall surgical satisfaction and people's general well-being."<br>"Extra surgery is never good, it implies more time of work, less money earned, and more time until the end of it all. It has a lot of influence on the investment made and approached surgical goal."
4	Post-operative bleeding	3·76 (1·04)	4 (3-5)	"I's a general risk of surgery; not only gGAS, and it can be managed effectively."<br>"It usually doesn't have effect on the long term outcomes of the genital surgery (functional or aesthetic)."<br>"It can be a bit scary when it happens and you need emergency surgery, so that can have impact on your mental health."
5	Wound dehiscence	3·97 (0·92)	4 (3-5)	"This isn't nice to have, but I've heard many people have this to some amount. Mostly it can be managed, but in some cases it can influence the aesthetics in the long run. I think there should be better info available to avoid this."<br>"I was acceptive of the risk, as long as it is treatable. I was still afraid it would impact my phallo length, but ended up having it only at my donor site so I was okay with that."

				"In my healing, I feel I wasn't properly prepared and knowledgeable to adequately help me through severe wound separation. During the revision stage I was very focused on fixing the tension around my wounds. I feel there could be more research into methods to prevent tension on the wounds."<br>"This is a common complication, but generally can be managed well. It does prolong the recovery period a bit."
6	Delayed wound healing	3·64 (1·04)	4 (3-4)	"It's important to pay attention to, also in regards to the recovery planning. In the end it's not as important as it heals fully and well. I rather not have this, but slow healing is still healing."<br>"A lot of people deal with this. It depends on why the healing is delayed, some reasons can also affect the aesthetics of scars. You can probably get that fixed in the long run, but that means another surgery and recovery period."<br>"The recovery period can already be tough emotionally. Delayed healing can be a setback, especially if it impacts daily life, like school and work."<br>"Almost every patient has this to some degree, in most cases it doesn't affect the end result."
7	Wound infection	4·02 (0·97)	4 (3,75-5)	"When I was thinking about this risk, I was thinking how likely is it and if it happens how easy it is to treat. For most infections, antibiotics will be sufficient, but this still can cause stress and make the recovery period more impactful."<br>"I did not have this, but I had excellent wound care to avoid it. My care team was also on top of it. If this is different between surgeons and hospitals, this might be something to look into."<br>"It is important, due to possibility severity effects of an infection. It can change the shape and size of scars and even the phallus. Can be mentally tough to deal with."<br>"Infection is an important outcome, but it is a risk for every surgery, therefor is doesn't really help differentiate between certain genital surgical techniques. That's the reason that it isn't CORE for me."
8	Time until complete recovery	3·85 (1·08)	4 (3-5)	"Realistic expectations of recovery time is essential for informed decision making. Also, it is important for planning the surgery and recovery period, you need to take off time from work, have enough sick-days and a financial situation that provides room for all this."<br>"Quicker recovery time allows for faster return to daily life and is better for the emotional well-being. Long recovery time can depress people and limits their daily and social life."<br>"Can vary per individual, depends on many factors. Very subjective as well. One patient can be more careful and slow with building up activity and another patient can be up and running in no time, sometimes too fast even."<br>"This is the reason why I chose meta instead of phallo, I couldn't do multi-month recovery periods. I do understand why others view it as a worthy sacrifice."
9	Need for re-intervention	4·38 (0·81)	5 (4-5)	"This happened to me and it caused significant harm to my day-to-day mental health over a period of three years. The waiting in between is awful. Every follow-up procedure requires recovery time and it influences how the phalloplasty looks, feels and functions."<br>"I feel that surgeons tend to underreport/underestimate this to patients. It is important to really be aware of this risk before making a choice for type of surgery. It can also cause financial strain."<br>"An important outcome. Yet, patient tend to be equally satisfied with the end result after re-interventions as those who didn't need them."<br>"Could reflect surgical competence and surgical success rate."
10	Readmission	3·92 (1·04)	4 (3-5)	"I think it usually is related to another complication (like bleeding or an infection) that could be measured more directly through another outcome measure. I would be more interested in the reason for readmission."<br>"This would still be difficult to compare, because the tendency to re-admit varies per country and healthcare system."<br>"Can be stressful when this happens, scary or even a major mental set back."<br>"Readmission can be influenced by other factors than the surgery itself, you need to know if there are patient characteristics that are involved, like underlying diseases and general health."

11	Functional status of flap donor site	4·37 (0·88)	5 (4-5)	"You get prepared that some impact on function can be expected, they do take a lot of tissue. I didn't expect that outcomes of the donor site would be so long term. After 6 years I still have intermittent swelling near my donor site."<br>"This is one of the reasons why I chose a meta, because I am very physically active and didn't want to compromise on movement and functioning. I also know people who worked with their hands a lot and couldn't risk that, but had little specific information on it."<br>"Donor site outcomes are important when choosing between different techniques. It's not always possible that people get to choose between all of the options, it also depends on physical characteristics. In that case the information would still be valuable for patient education."
12	Unplanned perineal urethrostomy	4·13 (1·07)	4 (4-5)	"This would have been a devastating, dysphoria inducing outcome for me. I haven't heard many people having this, but those who did had it as a last resort repair effort."<br>"It sounds like a nightmare for those whose goal it was to be able to stand while peeing. I didn't know about this complication, so I wasn't worried about it. I think you have a higher risk for other urinary problems. But I think it would be good to have more information about the chances of this happening."<br>"It can have major impact, however, it is very rare. I find it surprising that it's included in this survey, haven't happened to me or my colleagues, but if this happens often somewhere else that needs to be investigated."<br>"An unplanned scrotal urethrostomy is more often performed as a temporary treatment for urethral fistulas or strictures. Perineal urethrostomy is not often done."
13	Ability to void in a standing position	4·36 (0·86)	5 (4-5)	"It was important when choosing surgery, but now I sit to pee most of the time anyway. Many cisgender men also sit to pee, definitely when they get older and it takes longer, so I come to see that it's normal and common for all men."<br>"Standing to pee enables me to experience gender alignment, but also to navigate any context without the risk of being outed as trans, and that also helps for safety. It's convenient as well."<br>"This is the main reason why people want urethral lengthening. It is essential to know how many surgeries are needed to achieve this, how many problems have occurred and what other consequences those problems and extra surgeries had. To make a decision about it, you need to be able to weigh the chances."<br>"Often people with urethral lengthening cannot pee standing up without dropping their pants to their ankles or standing in uncomfortable positions to limit getting pee on their clothing, or only when standing in the shower. That's in my opinion not a successful urethral lengthening, we need to get the facts on those nuances straight."
14	Neo-urethral stricture	4·36 (0·86)	5 (4-5)	"This is a very common outcome, it's good to follow up on this since it has a delayed onset." "Anything that could involve extra surgery should be closely monitored, it could be a strain to finance, social life and mental health."<br>"It was really painful and took years and multiple revisions to manage. I didn't expect that and had little information before on how to deal with it. If I were to consider surgery today, I would like to know the numbers on it."<br>"Identifying the incidence and management of such a serious and common complication may help surgeons adjust their approach, but could also guide post-operative teaching of primary care providers when patients are discharged from the hospital."<br>"I would argue that the presence of a stricture is not important, but rather only the presence of a clinically significant stricture. Only when obstructing the urinary flow, it could lead to problems."
15	Neo-meatal stenosis	4·10 (1·00)	4 (3-5)	"I had this and it was fairly easy to fix."<br>"It's an unfortunate complication, but in general much easier to treat than a urethral stricture or fistula. So it's rather important, but has no high priority."<br>"Depends on having symptoms, only important if symptomatic. It needs to result in obstructive peeing and subsequent problems in order for it to be relevant."
16	Neo-urethral fistula	4·36 (0·86)	5 (4-5)	"I'd rather not have this complication, and if it were both highly likely and difficult to treat it would have impacted my decision making. It is helpful to know whether a surgery was needed for treatment or self-resolving."<br>"I had one and it still has not closed successfully after surgical repair. It's frustrating as it causes spraying while peeing when standing. I have to wipe afterwards, which sucks and creates dysphoria and feels like a huge set back."<br>"I had many of these, it was horrible. The insolvable, embarrassing urine smell made it impossible for me to leave the house."<br>"It is important that ALL fistula's should be reported, not only those who need surgery. Those that spontaneously heal can take

				months to do so and can have impact on the daily life. May require frequent changing of clothing, wearing padding, a moist feeling and skin breakdown.”
17	Post-void dribbling of urine	3·72 (1·06)	4 (3-5)	“This happens to a lot of people, but it is also common for cisgender men.”<br>“It is annoying, but not much of a problem if I milk and wipe.”<br>“Relevant, but not core outcome. No amount of scientific research will prevent another patient from having this problem that needs urethral milking or other management. Good to have more data for information purposes.”<br>“If it's severe, it can have a big impact on daily life, having to wear padding can be triggering.”
18	Post-void residual volume of urine	3·70 (1·11)	4 (3-5)	“Only relevant when symptomatic, when it is can lead to post-voiding dribbling of urine or a urinary tract infection.”<br>“It is important to know what the urinary function is like, especially over time. If problems can develop over time it is important to monitor it.”<br>“You need an pre-operative measurement to interpret correctly. If you have that, it is a useful measurement to catch problems like strictures or a weak bladder.”<br>“Risk of pyelonephritis, hydronephrosis and renal dysfunction if urinary function decreases. If this persists, it can result in having to use a CAD or needing surgery.”
19	Mean urinary flow rate	3·29 (1·07)	3 (3-4)	“You need a pre and post op measure to objectify change. Change can be predictive of future problems with the urinary function, even before someone develops symptoms.”<br>“I don't care how long it takes me to pee. It wouldn't bother me as long as it doesn't impact my health or impact my life in some larger way.”<br>“The maximum flow rate is more relevant, this is the first urinary outcome that changes over time after surgery and could indicate problems in a relative early stage.”<br>“This is an outcome that is objective, it can provide transparent data for informed decision making if collected collectively. With a lot of data, we can better tell what is normal and what is not.”
20	Genitals matching gender identity	4·22 (1·06)	5 (4-5)	“This is the entire purpose of having genital surgery. It greatly reduces dysphoria. It feels central to my motivation for surgery.”<br>“It is a very demanding set of surgeries, requiring reserves of physical, mental and emotional energy. Lived experience of this outcome versus the expectations adds value to the discussion.”<br>“It broadly measures the purpose of the surgery, but on an individual, patient centered level.”<br>“For me this was the reason to undergo surgery, but if I would have known that I would still have all of these issues after surgery, I would have never taken the step.”
21	Feeling confident about genitals	4·15 (0·92)	4 (4-5)	“One of the main reasons why people get this surgery is to get more comfortable with their genitals, but confidence is less important than resolving distress and discomfort.”<br>“Confidence is good and fine, but more important is feeling neutral-to-positive, rather than negative. People can be insecure about their genitals, regardless of having had surgery.”<br>“This is depending on many other factors as well, although it's a goal of the surgery, it's difficult to measure as directly related to the surgery.”<br>“Sex is important to me, and feeling confident is a big part of the sexual experience. It's needed to sexually explore.”
22	Genital gender dysphoria	4·45 (0·83)	5 (4-5)	“It is a mismatch between what I experience my body to be and what my physical embodiment actually is. For some gender might play a part in this, but for me it's more about a sense of what my right sex is. Gender dysphoria related to genitals does not measure this for me, it's more nuanced than that.”<br>“This is hugely important, this is the whole reason why we put ourselves through all this pain and misery.”<br>“This is one of the main aims of genital surgery, best possible overall item to measure. This assesses someone's perception and is therefore key to determine effectiveness of treatment.”<br>“This implies that people need to have genital gender dysphoria in order to receive surgery. Not all people experience it, although it is common. Would prefer alignment of one's body.”

23	Willingness to undergo genital gender surgery again	3·89 (1·16)	4 (3-5)	“I always say 'if my dick would fall off today, I'd be in the airplane back to my surgeon in Serbia for another operation tomorrow'.”<br>“There are many factors involved. Many people may be unsatisfied with their outcome but might choose the same procedure, but with a different surgeon, which is what I would choose. That's different.”<br>“I know people who had many complications who would definitely choose the same surgery again, so it can really tell how satisfied someone is in the end.”<br>“It says more about the care that someone received, rather than about the surgical technique in particular. I doubt if it influences decision making. Great for evaluation of provided care.”
24	Regret of decision to undergo genital gender surgery	4·16 (1·12)	5 (4-5)	“This is negatively framed, I like the outcome about willingness to undergo surgery again better, that is a bit more nuanced and about the thought behind it. I have no regrets, even with all of the complications and problems that I've encountered.”<br>“Regret is a very hot topic of concern, we need to get this accurate and consistent across studies. But we need to use it wisely, it needs to be so we can advocate for rights and healthcare politically. Can be scary in the wrong hands.”<br>“I don't think we should be focusing research on regret. I think we have moved passed that a long time ago.”<br>“Only measure this when taking the motivations and reasons into account, we need to know what makes the regret.”
25	Regret of type of genital gender surgery undergone	4·01 (1·04)	4 (3-5)	“If I were making this decision now, I'd absolutely want to know whether someone else would have made a different choice in retrospect. This can be very helpful in decision making for new people. Would be very interested in urethral lengthening versus non-urethral lengthening and meta versus phallo.”<br>“There is a possibility that the patient was nog properly advised about what to expect from the particular genital surgery. But it is important to know why the chosen surgery led to feelings or regret and whether there was anything that could have been done differently to avoid this.”<br>“This can be tough, because you don't always have a choice. I would have preferred ALT, but my body was better suited for RFF due to my weight. But I like that it is a more nuanced form of regret.”<br>“Regret is a very complex concept and can represent different types and scenarios. It is too subjective to measure as a general outcome of surgery. It also changes over the course of life, due to personal growth and development.”
26	Satisfaction with donor site aesthetic result	3·88 (0·90)	4 (3-5)	“Wasn't a concern for me undergoing meta, but was one of the big reasons why I opted for meta and not phallo due to not wanting the awful scarring and risks associated.”<br>“It's complicated and very personal. It is part of the surgery, so you're going to end up with a scar. It changes over time and some can cover it with clothing and some tattoo over it.”<br>“It's always going to look like a very large, conspicuous skin graft. Great for Pride. Not great for daily life.”<br>“Lots of guys worry that certain donor site scar will become generally recognizable and will out them over time. Comments of others on it may also impact euphoria.”
27	Acceptability of donor site morbidity	3·86 (1·08)	4 (3-5)	“I wouldn't want to compromise the mobility of certain body parts. People use tools and need their arms and hands for that.”<br>“I have just accepted this as a part of the surgery. The goal is to acquire a surgically created penis, to some degree you have to sacrifice another body part.”<br>“It is an important long-term outcome. Directly after surgery it seems like a minor inconvenience, but over time it can become really annoying that you still have no feeling in your donor-site area. So it is important for pre-operative counselling.”<br>“This is a very in-depth outcome and not related to the genitals, although it can be part of the surgery, it is far away from measuring genital outcomes.”
28	Satisfaction with neo-genital aesthetic result	4·33 (0·88)	5 (4-5)	“This is especially important if aesthetics were the goal of the operation and not function. Would be interesting to look at the contrast of function and aesthetics.”<br>“While aesthetics is less important than function to me, I do want to be able to see my gender reflected in the appearance of my genitals.”<br>“The scrotum is the most 'normal-looking' part of this kind of surgery. I like that this outcome includes the scrotum and is not only focused on the penis.”<br>“It is important, both for self-image and presentation of gender to others. Ability to 'pass' without the need to reveal trans status. It also helps for sexual confidence.”

29	Satisfaction with neo-phallus aesthetic result	4·10 (1·00)	4 (4-5)	"I think it's important to ask something about aesthetics. I am neutral about asking about aesthetics of the phallus specifically, versus genitals as a whole."<br>"I think it is more important to ask for the whole result than the result of the penis in particular. It's more inclusive."<br>"Looks are a big part of it. I really wanted it to look like a cis-penis. At least to pass for it, for the sake of safety in locker-rooms."<br>"I think this is an important result, but also very subjective. The reliability of this outcome is dependent in examining in a validated way."
30	Surgical result matching expectations	4·09 (0·95)	4 (4-5)	"This is very important, but the question must also ask what the expectations were before surgery, and whether these were met after surgery, and if not; which ones weren't met."<br>"This is heavily dependent on whether someone's expectations were realistic to begin with. It also tells about how well people are informed and are able to make decisions that fit them and have realistic goals."<br>"It often turns out that, despite expectation management before surgery, the surgery gets underestimated and outcomes over expected. So this is also a key indicator on patient education."
31	Satisfaction with surgical results	4·19 (1·12)	5 (4-5)	"This is a very broad outcome, there are other outcomes that measure this more specifically. Moreover, it is shown to be a unreliable outcome measure."<br>"This is a vague outcome, it's unclear what is meant by it. Those who had phallo and meta typically strongly dislike having their genitals referred to as results. Rephrase or use more specific outcomes."<br>"I am overall enormously satisfied with my surgical results. But it took me a long time and a lot of surgeries to get there. My satisfaction is felt in a narrow frame of what I know was possible to achieve with the surgery I had and the body I have. Ask more grained questions on what exactly is satisfying."<br>"Overall wellbeing and gender dysphoria are greatly affected by this."
32	Erogenous sensibility in neo-phallus	4·36 (0·87)	5 (4-5)	"I think people fear that it will just be an appendage. It's a sexual organ and it can be frustrating if there is no sexual sensation in it."<br>"This is one of the few subjective outcomes measures that is clinically significant and has a major impact on quality of life as well as being a reflection of surgical success."<br>"I had excellent erogenous sensation after my first meta surgery. With each revision and re-do that sensation declined significantly. What was effortless before became effortful as a result of continual surgical intervention."<br>"Erogenous sensibility can be an important part of sexual function. But there is much more to it, like how it is stimulated and experienced. Mental stimulation of being sexually active with the neo-phallus can be of big influence."
33	Erogenous sensibility in the clitoral glans	3·98 (1·10)	4 (3-5)	"This is important to me because I had a meta, so it is the head of my penis. The primary thing I use my penis for is sex."<br>"I wonder if it depends if it's buried or unburied with a phallo, but most importantly it's about if you're still able to orgasm, and usually people need their (clitoral) glans for that."<br>"The erogenous sensibility of the clitoral glans depends on the type of surgery."<br>"It rarely disappears. It would be more relevant to look at the sensation in the penis and the ability to orgasm."
34	Ability to perform sexual function as desired	4·32 (0·84)	4 (4-5)	"I appreciate that this outcome does not assume certain goals for everyone, however, it is so broadly worded that it is difficult to answer or to interpret data about it."<br>"People have different desires and need specific data on those desired sexual functions to make an informed decision."<br>"Important with orgasm and sensation, but also the ability for foreplay and creative use of your penis during intercourse."<br>"It's important but there are many confounding factors that get noise in the answers to this question. It is too broad."

35	Ability to achieve orgasm	4·29 (0·90)	5 (4-5)	"Sexual function preservation is important, the ability to achieve orgasm is probably the most important outcome to most people."<br>"The desire to orgasm varies from person to person, but is essential to most. Many people are also afraid to lose the ability after surgery. Having more information on it would help the distress and fear."<br>"It depends on whether someone also orgasmed before surgery and other factors are of influence as well (psychological/sexological)."<br>"For metoidioplasty the expectation is that ability to achieve orgasm is fully preserved, and many people choose this type of surgery because they do not want to risk any loss of function."
36	Ability to perform penetrative sexual intercourse	3·98 (1·08)	4 (3-5)	"Very important in my personal experience, but not as important to others, this is a variable desire amongst the population and should be addressed as such."<br>"This was the number one reason why I wanted a phalloplasty. I'm gay and not a bottom and I hated being treated like one."<br>"Having penetrative sex is something I always wanted to do. I wanted to feel it with my own anatomy."<br>"This is not the most important sexual function outcome, sensibility and ability to orgasm are more important to most people."
37	Satisfaction with sexual function	4·28 (0·84)	4 (4-5)	"While important, this outcome is somewhat vague and perhaps better measured by breaking out; achieving orgasm, ability to penetrate, ways to use penis during sex, sensation in the penis, engaging in kink activities etc."<br>"People have different desires, so to answer those specific desires the question needs to be specified. This is too broad and doesn't say much. It doesn't help understanding the nuances."<br>"This is important to many people, it is central to sexual well-being and quality of life."<br>"This is very personal and thus subjective, many other factors are of influence. This is more than just the function of the 'new penis'."
38	Overall sexual satisfaction	4·13 (0·89)	4 (4-5)	"This is a broad category, but it does give an indication of the degree of success the surgery gives for people in terms of sexual function. Therefor it may be useful. But for individual decision making it's not specific enough. It's only partly directly influenced by the surgery."<br>"The overall picture is more important to me than the specific functions. Important to sexual well-being and gender dysphoria."<br>"It's important to measure, but needs to be followed up by what exactly determines the sexual satisfaction and to what degree the surgery was of influence."<br>"This is a good, all compassing assessment that leaves room for the patients to report satisfaction without disclosing their specific sexual practices."

LEE = Lived Experience Expert, PE = Professional Expert, SD = Standard Deviation, Q1 = Quartile 1, Q3 = Quartile 3

Chapter 2B

Results Phase 2: Building consensus on outcome priority - Round 2

Table 3. Results of Phase 2: e-Delphi survey round 2

Survey round 1 order	Survey round 2 order	Outcome	LEE vote 1 or 2 (%)	LEE vote 4 or 5 (%)	Verdict LEE	PE vote 1 or 2 (%)	PE vote 4 or 5 (%)	Verdict PE	Combined verdict	Mean of combined ratings (SD)
3	1	Flap necrosis of the neo-phallus	17·6	82·4	NC	3·6	83·6	IN	NC	4·26 (1·05)
22	2	Genital gender dysphoria	29·4	61·8	NC	18·2	69·1	NC	NC	3·70 (1·32)
2	3	Tactile sensibility in the neo-phallus	17·6	70·6	NC	5·5	70·9	NC	NC	3·85 (1·02)
9	4	Need for re-intervention	14·7	61·8	NC	16·4	63·6	NC	NC	3·75 (1·07)
11	5	Functional status of flap donor site	14·7	79·4	IN	10·9	69·1	NC	NC	3·79 (0·99)
13	6	Ability to void in a standing position	17·6	67·6	NC	14·5	67·3	NC	NC	3·89 (1·23)
14	7	Neo-urethral stricture	11·8	73·5	NC	10·9	70·9	NC	NC	3·96 (1·09)
16	8	Neo-urethral fistula	11·8	85·3	IN	10·9	69·1	NC	NC	3·98 (1·03)
32	9	Erogenous sensibility in neo-phallus	8·8	73·5	NC	14·5	65·5	NC	NC	3·83 (1·04)
28	10	Satisfaction with neo-genital aesthetic result	2·9	91·2	IN	5·5	81·8	IN	IN	4·15 (0·86)
34	11	Ability to perform sexual function as desired	14·7	52·9	NC	12·7	65·5	NC	NC	3·79 (1·13)
35	12	Ability to achieve orgasm	14·7	67·6	NC	16·4	60·0	NC	NC	3·66 (1·03)
37	13	Satisfaction with sexual function	17·6	64·7	NC	9·1	63·6	NC	NC	3·78 (1·07)
20	14	Genitals matching gender identity	23·5	52·9	NC	21·8	56·4	NC	NC	3·57 (1·21)
31	15	Satisfaction with surgical results	20·6	61·8	NC	14·5	69·1	NC	NC	3·73 (1·11)
24	16	Regret of decision to undergo genital gender surgery	50·0	32·4	EX	30·9	50·9	NC	NC	3·19 (1·35)
21	17	Feeling confident about genitals	23·5	47·1	EX	32·7	32·7	EX	EX	3·09 (1·07)
12	18	Unplanned perineal urethrostomy	47·1	20·6	EX	36·4	45·5	EX	EX	3·04 (1·26)
38	19	Overall sexual satisfaction	17·6	52·9	NC	23·6	58·2	NC	NC	3·52 (1·17)
15	20	Neo-meatal stenosis	44·1	20·6	EX	29·1	41·8	EX	EX	2·99 (1·11)
29	21	Satisfaction with neo-phallus aesthetic result	17·6	52·9	NC	18·2	50·9	NC	NC	3·46 (1·03)
30	22	Surgical result matching expectations	29·4	47·1	EX	23·6	58·2	NC	NC	3·37 (1·13)
7	23	Wound infection	47·1	26·5	EX	30·9	49·1	EX	EX	3·12 (1·20)

25	24	Regret of type of genital gender surgery undergone	47·1	26·5	EX	36·4	43·6	EX	EX	3·06 (1·29)
33	25	Erogenous sensibility in the clitoral glans	35·3	29·4	EX	30·9	41·8	EX	EX	3·09 (1·11)
36	26	Ability to perform penetrative sexual intercourse	35·3	26·5	EX	30·9	43·6	EX	EX	3·04 (1·18)
5	27	Wound dehiscence	38·2	23·5	EX	30·9	36·4	EX	EX	3·02 (1·07)
10	28	Readmission	41·2	23·5	EX	29·1	38·2	EX	EX	3·01 (1·13)
23	29	Willingness to undergo genital gender surgery again	38·2	32·4	EX	23·6	50·9	NC	NC	3·26 (1·28)
26	30	Satisfaction with donor site aesthetic result	44·1	29·4	EX	27·3	47·3	EX	EX	3·09 (1·07)
27	31	Acceptability of donor site morbidity	32·4	35·3	EX	34·5	47·3	EX	EX	3·11 (1·08)
8	32	Time until complete recovery	35·3	29·4	EX	23·6	45·5	EX	EX	3·15 (1·11)
4	33	Post-operative bleeding	52·9	11·8	EX	34·5	40·0	EX	EX	2·81 (1·20)
1	34	Length of the neo-phallus	41·2	26·5	EX	52·7	25·5	EX	EX	2·71 (1·19)
17	35	Post-void dribbling of urine	44·1	17·6	EX	32·7	34·5	EX	EX	2·88 (1·11)
18	36	Post-void residual volume of urine	44·1	26·5	EX	40·0	41·8	EX	EX	2·98 (1·25)
6	37	Delayed wound healing	50·0	20·6	EX	45·5	34·5	EX	EX	2·75 (1·06)
19	38	Mean urinary flow rate	44·1	29·4	EX	40·0	36·4	EX	EX	2·90 (1·20)

LEE = Lived Experience Expert, PE = Professional Expert, SD = Standard Deviation, NC = No Consensus, IN = Included, EX = Excluded

Chapter 2C

Results Phase 2: Building consensus on outcome priority - Round 3

Table 4. Results of Phase 2: e-Delphi survey round 3

Survey round 2 order	Survey round 3 order	Outcome	LEE vote 1 or 2 (%)	LEE vote 4 or 5 (%)	Verdict LEE	PE vote 1 or 2 (%)	PE vote 4 or 5 (%)	Verdict PE	Combined verdict	Mean of combined ratings (SD)	Classification for phase 3
1	1	Flap necrosis of the neo-phallus	12·9	80·6	IN	7·5	84·9	IN	IN	4·25 (1·03)	Included
8	2	Neo-urethral fistula	16·1	77·4	NC	5·7	90·6	IN	NC	4·23 (1·03)	Included
7	3	Neo-urethral stricture	12·9	67·7	NC	3·8	92·5	IN	NC	4·20 (1·00)	No consensus
6	4	Ability to void in a standing position	22·6	77·4	NC	13·2	54·7	NC	NC	3·86 (1·09)	No consensus
3	5	Tactile sensibility in the neo-phallus	6·5	74·2	NC	11·3	77·4	IN	NC	3·92 (0·96)	Included
9	6	Erogenous sensibility in neo-phallus	6·5	77·4	IN	17·0	64·2	NC	NC	3·82 (1·08)	No consensus
5	7	Functional status of flap donor site	6·5	67·7	NC	5·7	58·5	NC	NC	3·77 (0·90)	Consensus of moderate importance
11	8	Ability to perform sexual function as desired	22·6	51·6	NC	18·9	52·8	NC	NC	3·60 (1·16)	Borderline exclusion
13	9	Satisfaction with sexual function	12·9	54·8	NC	15·1	66·0	NC	NC	3·68 (1·03)	No consensus
4	10	Need for re-intervention	12·9	80·6	IN	9·4	64·2	NC	NC	3·90 (1·00)	No consensus
15	11	Satisfaction with surgical results	19·4	54·8	NC	15·1	67·9	NC	NC	3·69 (1·01)	Consensus of moderate importance
2	12	Genital gender dysphoria	12·9	61·3	NC	20·8	64·2	NC	NC	3·71 (1·18)	Consensus of moderate importance
12	13	Ability to achieve orgasm	16·1	74·2	NC	26·4	47·2	EX	NC	3·54 (1·15)	No consensus
14	14	Genitals matching gender identity	16·1	58·1	NC	18·9	50·9	NC	NC	3·49 (1·08)	Borderline exclusion
19	15	Overall sexual satisfaction	12·9	67·7	NC	13·2	56·6	NC	NC	3·62 (1·01)	No consensus
21	16	Satisfaction with neo-phallus aesthetic result	6·5	80·6	IN	20·8	49·1	EX	NC	3·63 (1·06)	Excluded
22	17	Surgical result matching expectations	22·6	48·4	EX	32·1	43·4	EX	EX	3·25 (1·07)	Excluded
29	18	Willingness to undergo genital gender surgery again	12·9	45·2	EX	26·4	43·4	EX	EX	3·31 (1·15)	Excluded

16	19	Regret of decision to undergo genital gender surgery	41·9	35·5	EX	34·0	50·9	NC	NC	3·18 (1·37)	Borderline exclusion

LEE = Lived Experience Expert, PE = Professional Expert, SD = Standard Deviation, NC = No Consensus, IN = Included, EX = Excluded

Chapter 3
Discussion

An appropriate choice of scope can determine the effectiveness of a COS. Thus, it is important to clearly define the scope of a COS, specifically focusing on certain intervention types. The broader the scope, the less applicable and relevant a COS might become. The current scope of the masculinizing gGAS COS is clearly defined yet intentionally broad. This broadness was deliberately chosen because trans and gender diverse individuals compare these procedures and their outcomes during surgical treatment decision-making. Thus, there is a basis of outcomes relevant to all procedures with some additional outcomes specific to each procedure. This modularity enables the masculinizing gGAS COS users to customize it for their study's scope.

In the literature, some COS developers recommend setting higher consensus criteria for inclusion in study Phase 2, compared to the methodologically typical threshold of 70%, to limit the number of outcomes included early in the consensus process.[1] Based on this recommendation, the inclusion threshold was raised to 75% in this study. This adjustment proved effective, as only two outcomes met the threshold, with two more nearly meeting it. Other than raising the threshold, the consensus criteria were defined according to standard methodology.

It is unclear what number of outcomes makes a feasible COS. The literature remains divided on the optimal number of outcomes for a COS and the point at which the number of outcomes begins to affect its uptake. Some studies have found no relationship between the number of outcomes and the implementation of the COS. However, others indicate that a COS should include between 5 and 15 outcomes.[2] Additionally, literature suggests that Core Domain Sets (CDS) should be limited to no more than five-seven domains.[3] Nevertheless, core domains encompass multiple outcomes instead of specific ones (e.g., life impact, recourse use, pathophysiological manifestations, and physical function), and it remains unclear how this CDS recommendation translates to core outcomes.

Nonetheless, a COS is defined as a minimum set of outcomes and should remain concise for feasibility. The feasibility of a COS is closely related to what Outcome Measurement Instruments (OMI) are used to measure the included outcomes. However, the current sequential COS development methodology (i.e., first determine the what. then the how, and when) only advises to be aware of the potential influence of the OMIs on the feasibility and uptake of the COS.[4] Yet, determining at what number of outcomes a COS should be restricted without weighing the potential burden of an OMI is guessing.

To facilitate consensus, methodology guidelines recommend feeding back the participants' ratings and ratings of both the stakeholder groups in subsequent e-Delphi survey rounds.[4] However, based on this study's results, this would be feeding back very similar feedback for every outcome since almost all participants rated them high. The SSG decided to present the outcomes in the order they were rated in the previous round instead, creating a more visual appearance of the likelihood of an outcome making it into the COS. Furthermore, it was felt that the quotes with reasoning for ratings were much more valuable to feedback. Getting participants to come closer together in their ratings must involve having participants better understand *why* ratings were given; this provides a much richer context to the ratings of other stakeholders. However, it should be acknowledged that collecting, analyzing, and feeding back the qualitative data was a time and workforce-consuming task, which may only be achievable for some COS developers.

The current study included several outcomes that were either similar, overlapping or oppositely phrased, aiming to identify which outcomes were preferred by participants. For example, *satisfaction with neo-genital aesthetic result* and *satisfaction with neo-phallus aesthetic result* were overlapping, while *genitals matching gender identity* and *genital gender dysphoria* were oppositely (i.e., positively and negatively) phrased. Similarly, *willingness to undergo genital gender surgery again* and *regret of decision to undergo genital gender surgery* represented opposite perspectives. For most of these outcomes, consensus on the preferred phrasing or concept was achieved during the process. However, while *satisfaction with neo-genital aesthetic result* was included early on, *satisfaction with neo-phallus aesthetic result* continued to receive high ratings, even though participants were informed about the already included outcomes. This suggests that participants may have continuously prioritized this outcome over others during rating, potentially influencing the voting results. To avoid this, future COS developers should either refrain from including similar or oppositely phrased outcomes or formulate additional consensus criteria when doing so.

COS developers often use the same consensus criteria for the Delphi Study and the consensus meeting.[5, 6] In this study, however, it was decided to lower the consensus meeting threshold and combine the stakeholder votes rather than treat them separately. The reason for doing so was based on LEE's moderate interest in participating in the consensus meeting after the third e-Delphi survey (i.e., eight interested participants). A potentially small sample of the stakeholder groups during the consensus meeting would not be representative, and it would be challenging to meet consensus criteria. Furthermore, the heterogeneity among the LEE participants should be acknowledged due to the broad scope of masculinizing gGAS.

Discussion references

1. De Meyer D, Kottner J, Beele H, Schmitt J, Lange T, Van Hecke A, et al. Delphi procedure in core outcome set development: rating scale and consensus criteria determined outcome selection. J Clin Epidemiol. 2019;111:23-31.
2. Hughes KL, Clarke M, Williamson PR. A systematic review finds Core Outcome Set uptake varies widely across different areas of health. J Clin Epidemiol. 2021;129:114-23.
3. Beaton D ML, Grosskleg S, Shea B, Tugwell P (editors). The OMERACT Handbook Version 2.1[updated April 2021]. OMERACT; 2021.
4. Williamson PR, Altman DG, Bagley H, Barnes KL, Blazeby JM, Brookes ST, et al. The COMET Handbook: version 1.0. Trials. 2017;18.
5. Fish R, Sanders C, Adams R, Brewer J, Brookes ST, DeNardo J, et al. A core outcome set for clinical trials of chemoradiotherapy interventions for anal cancer (CORMAC): a patient and health-care professional consensus. Lancet Gastroenterol Hepatol. 2018;3(12):865-73.
6. Munblit D, Nicholson T, Akrami A, Apfelbacher C, Chen J, De Groote W, et al. A core outcome set for post-COVID-19 condition in adults for use in clinical practice and research: an international Delphi consensus study. The Lancet Respiratory Medicine. 2022;10(7):715-24.
